# Disease staging of Alzheimer’s disease using a CSF-based biomarker model

**DOI:** 10.1038/s43587-024-00599-y

**Published:** 2024-03-21

**Authors:** Gemma Salvadó, Kanta Horie, Nicolas R. Barthélemy, Jacob W. Vogel, Alexa Pichet Binette, Charles D. Chen, Andrew J. Aschenbrenner, Brian A. Gordon, Tammie L. S. Benzinger, David M. Holtzman, John C. Morris, Sebastian Palmqvist, Erik Stomrud, Shorena Janelidze, Rik Ossenkoppele, Suzanne E. Schindler, Randall J. Bateman, Oskar Hansson

**Affiliations:** 1 Clinical Memory Research Unit, Department of Clinical Sciences Malmö, Lund University, Lund, Sweden; 2 Tracy Family Stable Isotope Labeling Quantitation (SILQ) Center, Washington University School of Medicine, St. Louis, MO USA; 3 Department of Neurology, Washington University School of Medicine, St. Louis, MO USA; 4 Eisai, Inc., Nutley, NJ USA; 5 Department of Clinical Science, Malmö, SciLifeLab, Lund University, Lund, Sweden; 6 Department of Radiology, Washington University School of Medicine, St. Louis, MO USA; 7 Charles F. and Joanne Knight Alzheimer Disease Research Center, Washington University School of Medicine, St. Louis, MO USA; 8 Memory Clinic, Skåne University Hospital, Malmö, Sweden; 9 Alzheimer Center Amsterdam, Neurology, Vrije Universiteit Amsterdam, Amsterdam UMC location VUmc, Amsterdam, The Netherlands; 10 Amsterdam Neuroscience, Neurodegeneration, Amsterdam, The Netherlands

**Keywords:** Diagnostic markers, Prognostic markers, Alzheimer's disease, Ageing

## Abstract

Biological staging of individuals with Alzheimer’s disease (AD) may improve diagnostic and prognostic workup of dementia in clinical practice and the design of clinical trials. In this study, we used the Subtype and Stage Inference (SuStaIn) algorithm to establish a robust biological staging model for AD using cerebrospinal fluid (CSF) biomarkers. Our analysis involved 426 participants from BioFINDER-2 and was validated in 222 participants from the Knight Alzheimer Disease Research Center cohort. SuStaIn identified a singular biomarker sequence and revealed that five CSF biomarkers effectively constituted a reliable staging model (ordered: Aβ42/40, pT217/T217, pT205/T205, MTBR-tau243 and non-phosphorylated mid-region tau). The CSF stages (0–5) demonstrated a correlation with increased abnormalities in other AD-related biomarkers, such as Aβ-PET and tau-PET, and aligned with longitudinal biomarker changes reflective of AD progression. Higher CSF stages at baseline were associated with an elevated hazard ratio of clinical decline. This study highlights a common molecular pathway underlying AD pathophysiology across all patients, suggesting that a single CSF collection can accurately indicate the presence of AD pathologies and characterize the stage of disease progression. The proposed staging model has implications for enhancing diagnostic and prognostic assessments in both clinical practice and the design of clinical trials.

## Abstract (web summary)

Salvadó et al. developed and validated a CSF-based staging model for sporadic Alzheimer’s disease, which accurately reflects biomarker and clinical changes, enhancing diagnostic and prognostic assessments of participants for clinical setting and trials.

## Main

Currently, more than 50 million people are affected by dementia, and this number is expected to more than double by 2050 (ref. ^1^). Alzheimer’s disease (AD) is the most common form of dementia, characterized by the accumulation of extracellular plaques containing amyloid-β (Aβ) and intracellular tau aggregates in the forms of tau tangles and neuropil threads^2^. Over the last two decades, the AD field has moved toward the use of biomarkers to support the diagnostic and prognostic workup rather than relying solely on clinical symptoms^3^. This has been made possible by advancements of imaging and fluid biomarkers that accurately track AD pathology in vivo. Given that the accumulation of pathology can take many years to decades^3^ before any clinical symptoms appear, the use of biomarkers is critical to ensuring an early and reliable detection of AD^4^. Key biomarkers may help to improve patient diagnosis, management and prognosis^5–8^. In addition, the use of AD biomarkers will be even more important when disease-modifying treatments become widely available^9–11^. In this context, a more sophisticated personalized medicine approach to AD, based on high-performing AD biomarkers, will become crucial to select the optimal participants for specific treatments and for enrollment in clinical trials.

In recent years, multiple cerebrospinal fluid (CSF) biomarkers targeting different pathophysiological mechanisms have been developed (see ref. ^4^ for a review). There has been an increasing interest in developing biomarkers for measuring tau species phosphorylated at different residues. Among the phosphorylated tau (p-tau) species, p-tau181 (refs. ^12–17^), p-tau217 (refs. ^12,13,15,18,19^) and p-tau231 (refs. ^15,20–22^) or the phosphorylation occupancies (defined as the ratio between the phosphorylated and non-phosphorylated mid region tau (np-tau) fragments) have been studied in depth and have shown strong associations with Aβ pathology and moderate associations with tau (as measured by both positron emission tomography (PET)^18,23^ and neuropathology^24,25^). These biomarkers have shown their utility in improving the diagnostic workup of AD and the prediction of disease progression^12,13,19,26,27^. Other biomarkers, such as p-tau205 or the occupancy (pT205/T205)^28–30^ and microtubule binding region (MTBR) of tau containing the 243 residue (MTBR-tau243)^31,32^, have been more closely related to tau tangle pathology. Importantly, some of these CSF biomarkers were shown to become abnormal at different phases during the progression of autosomal dominant Alzheimer’s disease (ADAD)^29^, suggesting a sequence of CSF biomarker changes that may serve as a measurable biological indicator tracking advancing disease progression.

The progression of Aβ or tau pathology across the brain has been previously used to stage participants across the AD continuum^33–38^. However, these models need at least one Aβ-PET or tau-PET scan, which is expensive and requires specialized personnel and facilities. Furthermore, information of only one pathological measure (for example, Aβ or tau) can be obtained from these images, and, therefore, they cover a limited range of the whole continuum. On the contrary, CSF biomarkers are less expensive and more accessible, and multiple pathological measures may be obtained from a single sample. Given this, and with the idea that different CSF biomarkers may become abnormal at different stages of the disease, we aimed to generate a data-driven staging scheme for sporadic AD using key CSF tau biomarkers in combination with CSF Aβ42/40. An unresolved question is whether there is a single molecular pathway throughout the AD continuum or whether there are subtypes of AD following different fluid biomarker trajectories, as has been shown for regional spread of insoluble tau tangles^36,39,40^.

In the present study, we used Subtype and Stage Inference (SuStaIn)^41^ to model the most likely sequence of CSF biomarker abnormalities that occur along the AD timecourse. This data-driven method uses cross-sectional data to order biomarker abnormalities in a probabilistic manner and, at the same time, addresses possible diverging trajectories of this ordering. Thus, we staged 426 participants of the Swedish BioFINDER-2 study, ranging from cognitively unimpaired (CU) participants to patients with mild cognitive impairment (MCI) or dementia, and compared to measures of AD pathology and progression. Finally, we replicated our results in an independent cohort (from the Charles F. and Joanne Knight Alzheimer Disease Research Center (Knight ADRC)), which included 222 participants.

## Results

A total of 426 participants from the Swedish BioFINDER-2 study (NCT03174938)^19^ with complete CSF data were included in the present study. Of these, 80 were cognitively unimpaired Aβ negative (CU−); 79 were cognitively unimpaired Aβ positive (CU+); 88 were diagnosed with MCI and were Aβ positive; 100 were diagnosed with AD dementia and were Aβ positive (ADD+); and 79 were assessed as non-AD patients (22 were Aβ positive). Demographic information is presented in Table 1 (see Supplementary Table 1 for demographic information by diagnostic groups). More detailed information about vascular risk factors and pathologies is provided in Supplementary Table 1, and a description of the diagnosis for non-AD patients can be found in Supplementary Table 2. Of these, 220 participants had longitudinal CSF data available (Supplementary Table 3).

**Table 1:** Participant characteristics

	BioFINDER-2 (*n* = 426)	Knight ADRC (*n* = 222)
Age, years	71.5 (8.5)	71.2 (7.7)
Women, *n* (%)	211 (49.5%)	112 (50.5%)
*APOE-ε4* carriership, *n* (%)^a^	246 (57.7%)	99 (44.6%)
Years of education^b^	12.3 (3.8)	16.3 (2.5)
Diagnosis, CU−/CU+/MCI+/ADD+/non-AD*CU−/CU+/Very mild AD/AD dementia/Other dementias**, *n*	80/79/88/100/79	84/98/24/9/7
Amyloid-PET, Centiloids^c^	37.3 (44.2)	44.0 (41.2)
Tau-PET, SUVR^d^	1.53 (0.61)	1.24 (0.22)
Cortical thickness, mm^e^	2.46 (0.16)	2.52 (0.16)
CSF NfL^f^	245 (175)	1000 (578)
Cognitive composite^#g^	−1.62 (2.03)	0.44 (1.11)
Progressed to MCI^†h^	11 (2.6%)	41 (18.5%)
Progressed to ADD+^¥i^	41 (9.6%)	30 (14.5%)

Data are shown as mean (s.d.) unless otherwise stated. * BioFINDER-2 participants are classified by clinical diagnosis and amyloid status based on their CSF Aβ42/40 levels (Aβ+: <0.080). ** Knight ADRC participants are classified by clinical diagnosis and amyloid status based on their CSF Aβ42/40 levels (Aβ+: <0.0673). In BioFINDER-2, only participants who progressed to MCI or patients with dementia due to AD etiology were considered to progress. In Knight ADRC, patients with very mild AD dementia had CDR = 0.5, and patients with mild AD dementia had CDR ≥ 1, both with AD as etiology. The ‘Other dementias’ group includes participants with CDR > 0 with non-AD etiology. Only participants who progressed to CDR ≥ 0.5 or CDR ≥ 1 due to AD etiology were considered to progress.

^#^Cognitive composite was mPACC for BioFINDER-2 and a global cognitive composite in Knight ADRC.

^†^For Knight ADRC, represents progression to CDR ≥ 0.5.

^¥^ For Knight ADRC, represents progression to CDR ≥ 1.

^a^One participant missing in both cohorts.

^b^Four participants missing in BioFINDER-2.

^c^One hundred seventy-five participants missing in BioFINDER-2.

^d^Nine and three participants missing in BioFINDER-2 and Knight ADRC, respectively.

^e^Six participants missing in BioFINDER-2.

^f^Four and five participants missing in BioFINDER-2 and Knight ADRC, respectively.

^g^Thirty-six and two participants missing in BioFINDER-2 and Knight ADRC, respectively.

^h^Four participants missing in Knight ADRC.

^i^Eight participants missing in Knight ADRC.

### CSF staging model

**Fig. 1:**
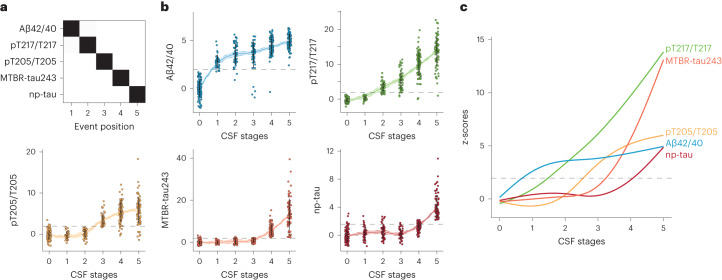
CSF staging model. Description of the CSF staging model and the levels of the biomarkers included in the model by CSF stage. Cross-validated confusion matrix of the CSF biomarkers of the model is shown in **a**. Biomarkers are sorted by the time they become abnormal based on the results of SuStaIn. Darkness represents the probability of that biomarker of becoming abnormal at that position, with black being 100%. Only amyloid-positive participants are included in this analysis. Individual biomarker levels by CSF stage in all BioFINDER-2 participants are shown in **b**. CSF levels are z-scored based on a group of CU− participants (*n* = 63), and all increases represent increase in abnormality. Colored lines and bands represent the LOESS regression and its 95% CI. Horizontal line is drawn at z-score = 1.96, which represents 95% CI of the reference group (CU−). Smoothed LOESS lines of all CSF biomarkers are shown in **c** for comparison. CSF stage 0 represents being classified as normal by the model. Black dots and vertical lines represent mean and 2 s.d. by CSF stage, respectively.

**Extended Data Fig. 1:**
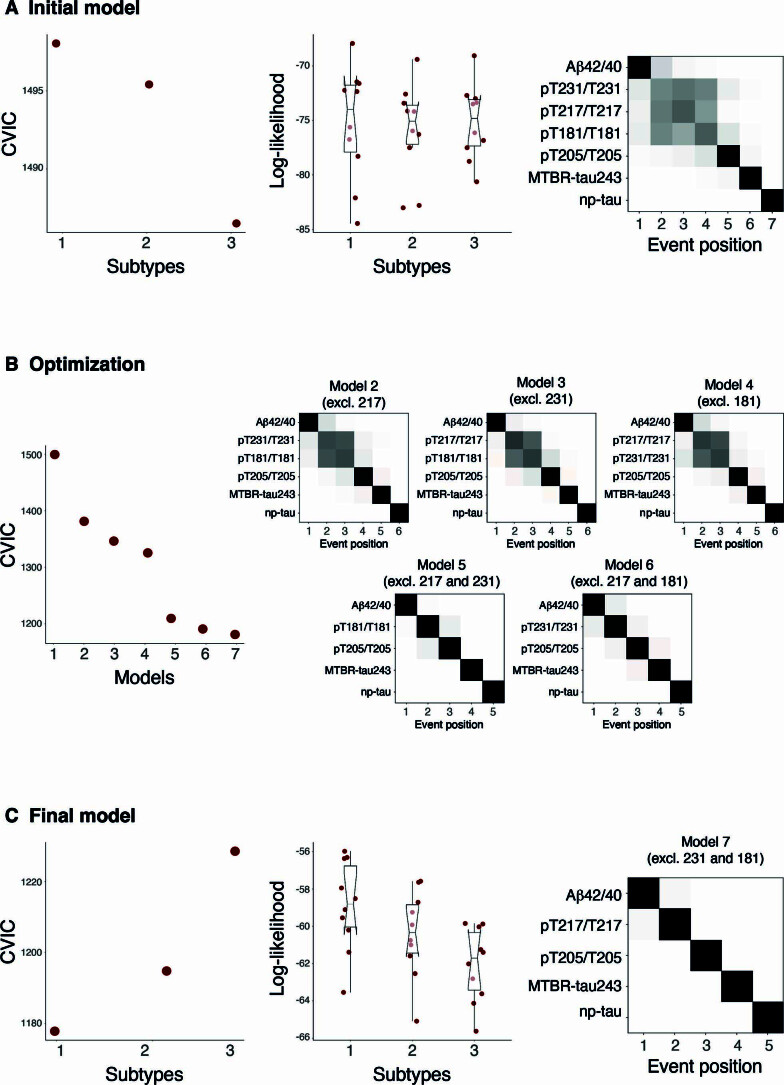
Creation and optimization of the model in the BioFINDER-2 cohort. Initial model with all CSF biomarkers (Aβ2/40, pT217/T217, pT231/T231, pT181/T181, pT205/T205, MTBR-tau243 and np-tau) is shown in A. First two columns represent the statistics, CVIC and log-likelihood, of this model for one, two and three subtypes. Each dot in log-likelihood plot represents one of the ten cross-validation sets of data. Lower CVIC and higher log-likelihood values represent better performance of the model. Although higher number of subtypes had higher CVIC, the comparable log-likelihood across subtypes suggests that one subtype is complex enough to explain the variability observed in the data. Cross-validated confusion matrix of the one subtype model is shown in the last column. Here, biomarkers are sorted by the time they become abnormal based on the results of SuStaIn. Darkness represents the probability of that biomarker of becoming abnormal at that position, with black being 100%. Given that some biomarkers (pT217/T217, pT231/T231 and pT181/T181) show high overlap on the ordering, we optimized the model by removing these biomarkers systematically (B). All models without one or two of these biomarkers were tested (models 2 to 7). CVIC (left) and cross-validated confusion matrixes (right) for each of these models are shown in B, respectively. CVIC shows that the optimal model was that excluding both pT231/T231 and pT181/T181 (model 7, shown in C). Both CVIC and log-likelihood measures show that one subtype was the optimal model when using this set of biomarkers. In boxplots, dots represent each of the ten-fold permutations, central band of the boxplot represents the median of the group, the lower and upper hinges correspond to the first and third quartiles, and the whiskers represent the maximum/minimum value or the 1.5 IQR from the hinge, whatever is lower. Abbreviations: Aβ, amyloid-β; CVIC, cross-validation information criterion; MTBR, microtubule binding region; np-tau, non-phosphorylated mid-region tau; pT, phosphorylated tau; SuStaIn, subtype and stage inference.

**Extended Data Fig. 2:**
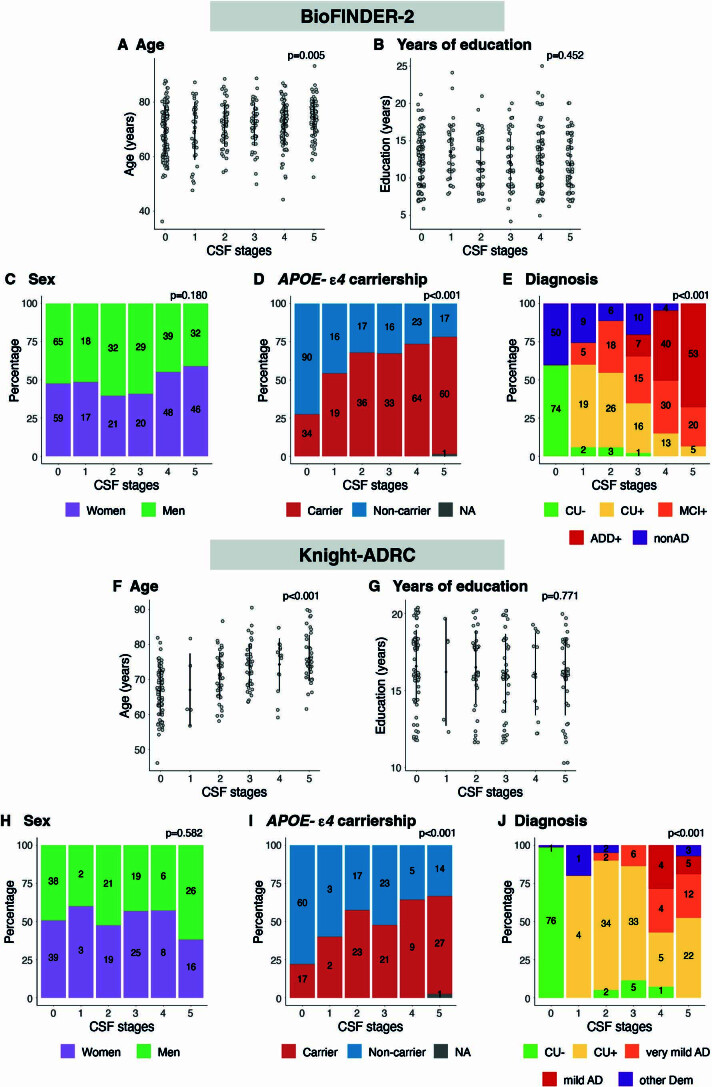
Demographic, genetic and clinical characteristics by CSF stage. Depiction of basic characteristics of BioFINDER-2 (A-E) and Knight-ADRC (F-J) by CSF stage. Kruskal-Wallis or chi-square tests were used to investigate the association between each of these characteristics and CSF stages. Two-sided p-values of these tests are shown at the top right of each subplot. Number of individuals in each category are shown inside the barplots. Black central dot and vertical lines in A, B, F and G represent the mean and two standard deviations of each stage, respectively. Abbreviations: AD, Alzheimer’s disease; ADD+, Alzheimer’s disease dementia amyloid positive; CU-, cognitively unimpaired amyloid negative; CU+, cognitively unimpaired amyloid positive; CSF, cerebrospinal fluid; MCI+, mild cognitive impairment amyloid positive; nonAD, non-Alzheimer’s related disease; other Dem, non-Alzheimer’s type dementia.

We initially applied SuStaIn to the BioFINDER-2 cohort using the following CSF biomarkers: the Aβ42/40 ratio, the phosphorylated to np-tau ratio of pT205/T205, pT181/T181, pT217/T217 and pT231/T231 as well as the concentrations of MTBR-tau243 and np-tau (the residue 151–155) based on availability and previous literature. Of note, the np-tau is different than the total-tau measures typically used in the clinical setting, which include both phosphorylated and np-tau fragments. Through a process of model optimization (Extended Data Fig. 1; see Methods for further details), we arrived on a model that excluded pT181/T181 and pT231/T231 due to information redundancy. SuStaIn revealed that a single biomarker sequence best described the progressive abnormality of the selected biomarkers (Extended Data Fig. 1c). The final ordering of the model was the Aβ42/40 ratio, pT217/T217, pT205/T205, MTBR-tau243 and np-tau (Fig. 1a), resulting in a five-stage model (plus stage 0 as a negative biomarker stage). Of note, the one-subtype model fit the data best even before performing the optimization step with all fluid biomarkers (Extended Data Fig. 1a). All BioFINDER-2 participants were then classified into one of these biomarker-based disease stages based on their CSF levels, with 124 (29.1%) being at CSF stage 0, 35 (8.2%) being at CSF stage 1, 53 (12.4%) being at CSF stage 2, 49 (11.5%) being at CSF stage 3, 87 (20.4%) being at CSF stage 4 and 78 (18.3%) being at CSF stage 5. Demographic, genetic and diagnostic characteristics of these participants are shown in Extended Data Fig. 2. In brief, the CSF biomarker-based model was not associated with sex (χ^2^(5) = 7.7, *P* = 0.180) or years of education (χ^2^(5) = 4.7, *P* = 0.452), but higher CSF stage was associated with older age (χ^2^(5) = 16.9, *P* = 0.005), carriership of an *APOE-ε4* allele (χ^2^(5) = 72.8, *P* < 0.001) and a more advanced clinical disease stage (χ^2^(5) = 478.6, *P* < 0.001) (Extended Data Fig. 2a–e).

We then examined the distribution of the CSF biomarkers included in the model by CSF biomarker stage. CSF biomarker levels by stage can be found in Fig. 1b and Supplementary Table 4. These different biomarker trajectories revealed that the included CSF biomarkers exhibit different behaviors across the disease continuum, aside from the biomarker disease stage at which they become abnormal. This is summarized in Fig. 1c, in which the smoothed locally estimated scatterplot smoothing (LOESS) regression of all CSF biomarkers is plotted. We found that none of the vascular risks (hypertension, hyperlipidemia or diabetes) nor vascular pathologies (white matter lesions, lacunes, ischemic infarcts, hemorrhages, microbleeds or siderosis) have an effect on our model (Supplementary Table 1 and Supplementary Figs. 1 and 2).

**Extended Data Fig. 3:**
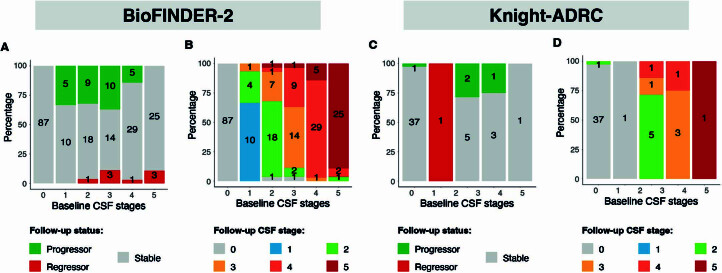
Model stability. Depiction of the evolution of CSF stages in BioFINDER-2 (n = 220, A-B) and Knight-ADRC participants (n = 51, C-D) with longitudinal CSF available. As longitudinal CSF Aβ42/40 levels were not available for any BioFINDER-2 participant, we imputed this data with their baseline levels. We show the number of progressors, regressors and stable participants in A and C, for each cohort respectively. In B and D, we further show the CSF stages at follow-up. For those Knight-ADRC with more than one longitudinal visit we took the one more distant from the baseline. Abbreviations: Aβ, amyloid-β; CSF, cerebrospinal fluid.

Finally, we assessed the stability of our model using the longitudinal CSF data over a mean (s.d.) of 2.1 (0.2) years (*n* = 220; Supplementary Table 1). We observed that most participants remained at the same stage (*n* = 183, 83.2%) or progressed (*n* = 29, 13.2%), whereas only few regressed (*n* = 8, 2.9%) (Extended Data Fig. 3a,b). Of those who progressed, most (*n* = 25, 86.2%) progressed only one CSF stage during the 2-year follow-up. This indicates a high stability of our model over time. Of note, participants with longitudinal CSF information had lower levels of pathology as measured by main biomarkers than those without longitudinal CSF data, even while having similar demographic characteristics (Supplementary Table 5).

### Associations with AD pathology, biomarkers and cognition

**Fig. 2:**
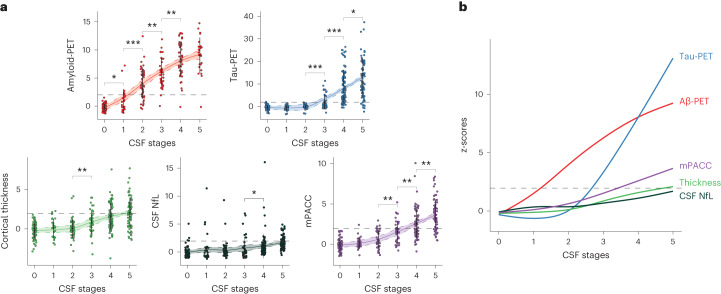
AD pathology biomarkers and cognition by CSF stages. **a**, Depiction of individual biomarker levels, not used in the creation of the model, by CSF stage in BioFINDER-2 participants. These include biomarkers of amyloid (amyloid-PET) and tau (tau-PET in the meta-temporal ROI) pathologies, neurodegeneration (cortical thickness in the AD signature areas and CSF NfL) and cognition (mPACC). Biomarkers are z-scored based on a group of CU− participants (*n* = 63), and all increases represent increase in abnormality. Significant differences in contiguous CSF stages are shown with asterisks (two-sided, FDR-corrected). The horizontal line is drawn at z-score = 1.96, which represents 95% CI of the reference group (CU−). Colored lines and bands represent the LOESS regression and its 95% CI. Smoothed LOESS lines of all AD biomarkers are shown in **b** for comparison. All participants with available data were included in amyloid-PET and tau-PET analyses. For neurodegeneration (cortical thickness and NfL) and cognitive (mPACC) measures, we excluded patients with non-AD dementia to avoid bias. Of note, only few AD dementia cases had amyloid-PET available due to study design. CSF stage 0 represents being classified as normal by the model. Black dots and vertical lines represent mean and 2 s.d. per CSF stage, respectively. *P < 0.05; **P < 0.01; ***P < 0.001. Exact *P* values shown in the figure are as follows. Amyloid-PET: 0–1, *P* = 0.032; 1–2: *P* = 1.6 × 10^−6^; 2–3: *P* = 0.003; 3–4: *P* = 0.0007. Tau-PET: 2–3: *P* = 0.0003; 3–4: *P* = 3.3 × 10^−11^; 4–5: *P* = 0.010. Cortical thickness: 2–3: *P* = 0.006. CSF NfL: 3–4: *P* = 0.016. mPACC: 2–3: *P* = 0.004; 3–4: *P* = 0.002; 4–5: *P* = 0.0008.

Next, we investigated the association between CSF stages and insoluble Aβ aggregates (Aβ-PET), insoluble tau aggregates (tau-PET), neurodegeneration (cortical thickness and CSF neurofilament light (NfL)) and cognition, using a global cognitive composite sensitive to early AD changes (modified version of Preclinical Alzheimer’s Cognitive Composite (mPACC)^35^; Fig. 2). The degree of biomarker abnormality increased with higher CSF stages, although the trajectories were different. Statistics of each of these AD biomarkers and their differences per CSF stage can be found in Supplementary Table 6.

**Fig. 3:**
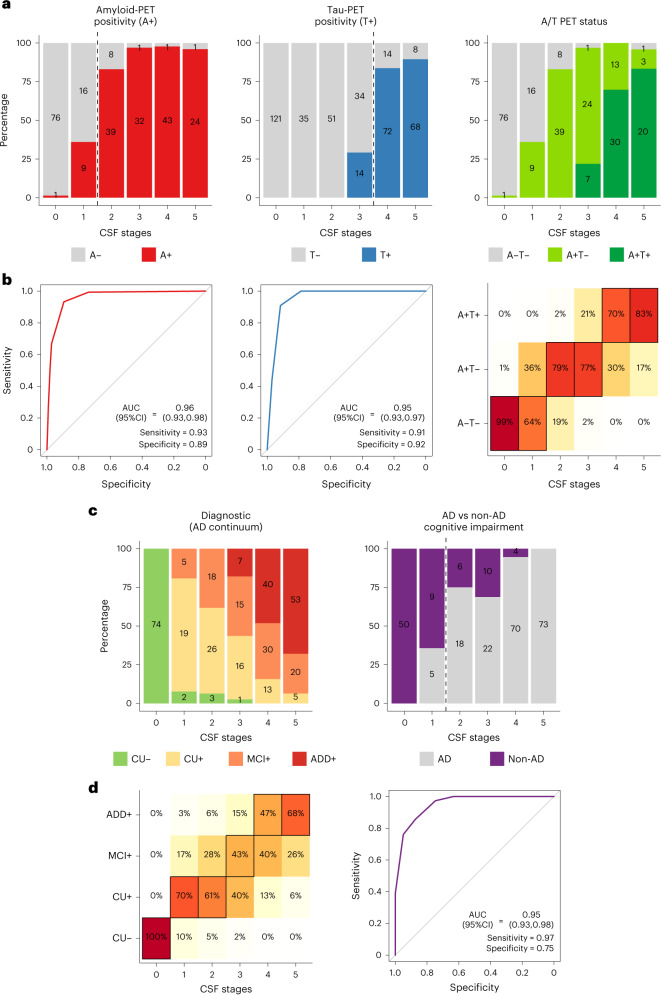
CSF stages for predicting A/T status and cognitive stages. CSF stages for predicting pathological status as measured with PET are shown in **a** and **b** and for predicting cognitive stages and diagnostic groups in **c** and **d**. Bar plots represent the number of participants in each category per CSF stage. Numbers of participants in each category per CSF stage are shown within the bar plots (**a** and **c**). In **b** and **d**, ROC curves were used to assess the classification into dichotomic categories (Aβ-PET, tau-PET and AD versus non-AD cognitive impairment), whereas ordinal logistic regressions were used for ordinal categories (A/T status and diagnosis). Heat maps represent the predicted percentage of participants in each outcome category (A/T or diagnosis) by CSF stage. The most probable (highest percentage) category by CSF stage is framed in black. For ROC analyses, AUCs and sensitivity and specificity measures from these analyses are shown in the plot. The optimal cutoff in each case is shown as a vertical dashed line in **a** or **c**. An A−T+ participant (*n* = 1) was excluded from the A/T status analysis. Non-AD dementia cases were excluded from the cognitive stages analysis. In addition, only patients with objective impairment (MCI or dementia) were included in the analyses of AD versus non-AD. Amyloid-PET and tau-PET were assessed as positive based on previously validated cutoffs (amyloid-β: SUVR > 1.03, tau: SUVR > 1.36).

**Extended Data Fig. 4:**
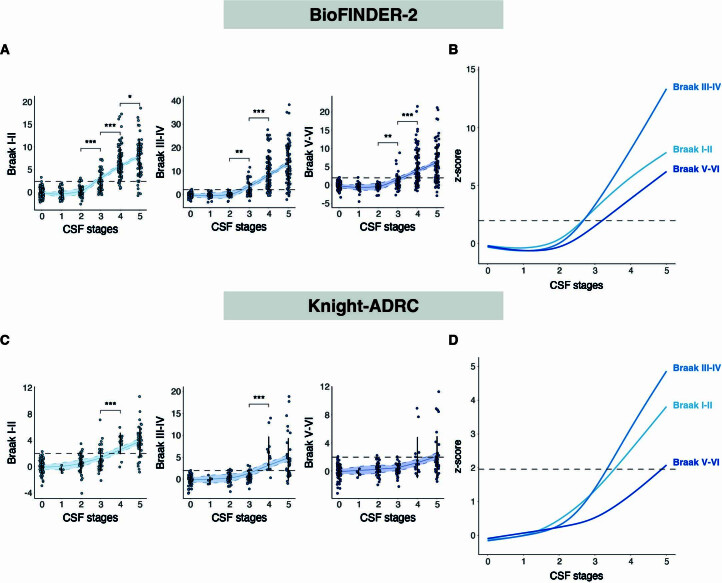
Tau-PET binding in different Braak regions by CSF stages. Depiction of tau-PET binding in different areas of tau deposition, by CSF stage in all BioFINDER-2 (A) and Knight-ADRC participants (B). These areas include regions of early (Braak I-II), intermediate (Braak II-IV) and late (Braak V-VI) tau deposition. Tau-PET levels are z-scored based on a group of CU- participants (BioFINDER-2: n = 63 and Knight-ADRC: n = 71) and all increases represent increase in abnormality. Significant differences in contiguous CSF stages are shown with asterisks (two-sided, FDR-corrected). Horizontal line is drawn at z-score = 1.96 which represents 95%CI of the reference group (CU-). Black central dot and vertical lines in A, and C represent the mean and two standard deviations of each stage, respectively. Colored lines and bands represent the LOESS regression and its 95%CI. Smoothed LOESS lines of all AD biomarkers are shown in B (BioFIDNER-2) and D (Knight-ADRC) for comparison. CSF stage 0 represent being classified as normal by the model. *: p < 0.05; **: p < 0.01; ***: p < 0.001. Exact p-values shown in the figure are, Braak I-II: 2-3: p = 5.8·10^−7^; 3-4: p = 9.2·10^−13^; 4-5: p = 0.041; Braak III-IV: 2-3: p = 0.0007; 3-4: 3-4: p = 5.8·10^−11^; Braak V-VI: 2-3: p = 0.0005; 3-4: p = 6.3·10^−7^; for BioFINDER-2; and: Braak I-II: 3-4: p = 1.4·10^−6^; Braak III-IV: 3-4: p = 5.2·10^−10^; for Knight-ADRC. Abbreviations: Aβ, amyloid-β; AD, Alzheimer’s disease; CI, confidence interval; CU-, cognitively unimpaired amyloid negative; CSF, cerebrospinal fluid; LOESS, locally estimated scatterplot smoothing; PET, positron emission tomography; ROI, region of interest.

**Extended Data Fig. 5:**
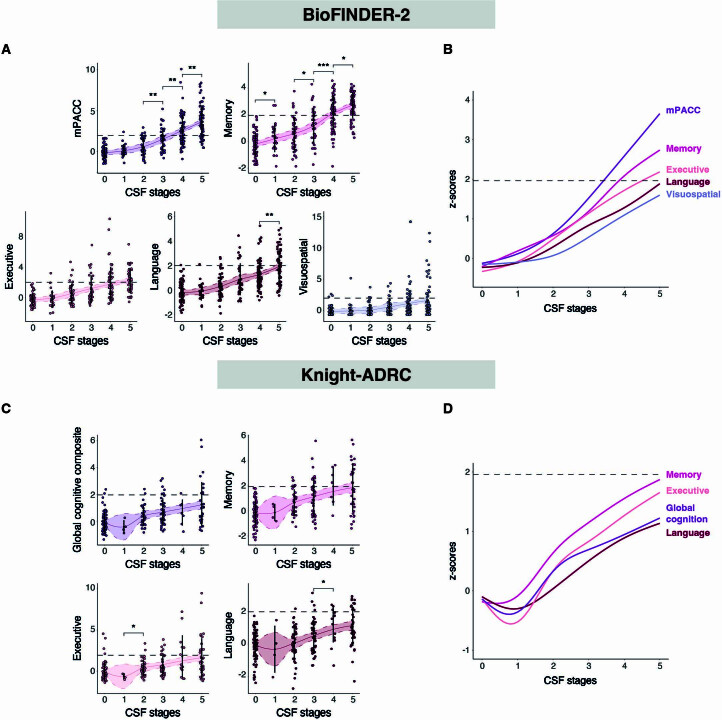
Cognitive composites by CSF stages. Depiction of different cognitive measures, by CSF stage in BioFINDER-2 (A) and Knight-ADRC participants (B). These measures include: mPACC (ADAS-delayed, animal fluency, MMSE and TMT-A), memory (ADAS-delayed and ADAS-immediate), executive function (TMT-A, TMT-B and symbols digit), language (animal fluency and BNT-15) and visuospatial (VOSP-cube and VOSP-incomplete) for BioFINDER-2. For Knight-ADRC we had a global cognitive composite (FCSRT, animals, TMT-A and TMT-B), an executive function composite (TMT-A and TMT-B), a memory (FCSRT) and language (animal fluency) tests. Cognitive scores are z-scored based on a group of CU- participants (BioFINDER-2: n = 60 and Knight-ADRC: n = 71) and all increases represent increase in abnormality. Significant differences in contiguous CSF stages are shown with asterisks (two-sided, FDR-corrected). Horizontal line is drawn at z-score = 1.96 which represents 95%CI of the reference group (CU-). Black central dot and vertical lines in A and C represent the mean and two standard deviations of each stage, respectively. Colored lines and bands represent the LOESS regression and its 95%CI. Smoothed LOESS lines of all AD biomarkers are shown in B (BioFIDNER-2) and D (Knight-ADRC) for comparison. We excluded non-AD dementia patients to avoid bias in these analyses. CSF stage 0 represents being classified as normal by the model. *: p < 0.05; **: p < 0.01; ***: p < 0.001. Exact p-values shown in the figure are, mPACC: 2-3: p = 0.004; 3-4: p = 0.002; 4-5: p = 0.0008; Memory: 0-1: p = 0.049; 2-3: p = 0.045; 3-4: p = 0.0002; 4-5: p = 0.011; Language: 4-5: p = 0.001; for BioFINDER-2; and Executive: 1-2: p = 0.044; Language: 3-4: p = 0.019 for Knight-ADRC. Abbreviations: AD, Alzheimer’s disease; ADAS, Alzheimer’s disease assessment scale; BNT, Boston naming test; CI, confidence interval; CU-, cognitively unimpaired amyloid negative; CSF, cerebrospinal fluid; FCSRT, free and cued selective reminding test; LOESS, locally estimated scatterplot smoothing; MMSE, Mini-Mental state examination; mPACC, modified version of preclinical Alzheimer’s disease cognitive composite; TMT, trial making test; VOSP, visual object and space perception battery.

We further studied the associations between our CSF-based staging model and other biomarkers as additional analyses. For tau-PET, we quantified the signal in different brain regions, using the previously validated regions of interest (ROIs) reflecting the different Braak stages^42^ (Extended Data Fig. 4 and Supplementary Table 7). We also examined different measures of cognitive function, including composites for memory, executive, language and visuospatial functions, respectively (Extended Data Fig. 5 and Supplementary Table 8).

**Fig. 4:**
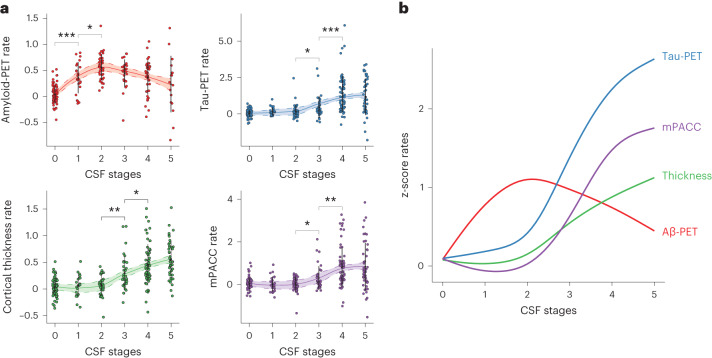
Longitudinal rate of change of AD biomarkers by CSF stages. **a**, Depiction of individual biomarker longitudinal rates of change by CSF stage in BioFINDER-2 participants. These include biomarkers of amyloid (amyloid-PET) and tau (tau-PET in the meta-temporal ROI) pathologies, neurodegeneration (cortical thickness in the AD signature) and cognition (mPACC). Biomarkers are z-scored based on a group of CU− participants (*n* = 63), and all increases represent increase in abnormality. Rates of change were calculated with individual linear regression models. Significant differences in contiguous CSF stages are shown with asterisks (two-sided, FDR-corrected). Colored lines and bands represent the LOESS regression and its 95% CI. Smoothed LOESS lines of all AD biomarkers are shown in **b** for comparison. All participants were included in amyloid-PET and tau-PET analyses. For neurodegeneration (cortical thickness) and cognitive (MMSE) measures, we excluded patients with non-AD dementia to avoid bias. CSF stage 0 represents being classified as normal by the model. Black dots and vertical lines represent mean and 2 s.d. per CSF stage, respectively. *P < 0.05; **P < 0.01; ***P < 0.001. Exact *P* values shown in the figure are as follows. Amyloid-PET: 0–1, *P* = 8.4 × 10^−5^; 1–2: *P* = 0.025. Tau-PET: 2–3: *P* = 0.032; 3–4: *P* = 4.6 × 10^−5^. Cortical thickness: 2–3: *P* = 0.001; 3–4: *P* = 0.041. mPACC: 2–3: *P* = 0.019; 3–4: *P* = 0.003.

### Prediction of Aβ/tau status and cognitive stages

Subsequently, we looked at the accuracy of our CSF staging model for predicting Aβ (A) and tau (T) status, as defined by PET^34^ (Fig. 3a,b). We first looked at each independent pathology dichotomously (that is, positive or negative) and independently, and, later, we looked at the ordinal categories merging both pathologies (that is, A−T−, A+T− and A+T+). The number of positive participants by CSF stage and category are presented in Fig. 3a. Using receiver operating characteristic (ROC) curve analyses, we determined that CSF stage 2 was the optimal threshold for predicting amyloid-PET positivity with high accuracy (area under the curve and 95% confidence interval (AUC (95% CI) = 0.96 (0.93, 0.98), sensitivity = 0.93 and specificity = 0.89, first column; Fig. 3b and Supplementary Table 9)). Tau-PET positivity was also predicted with high accuracy when using CSF stage 4 as a threshold (AUC (95% CI) = 0.95 (0.93, 0.97), sensitivity = 0.91 and specificity = 0.92, second column; Fig. 3b and Supplementary Table 9).

Ordinal logistic regression was used to assess the utility of CSF stages for predicting A/T status (that is, A−T−, A+T− or A+T+), and we calculated the c-index (an overall measure of discrimination equivalent to AUC for dichotomic outcomes) as a measure of accuracy. We observed that higher CSF stages were associated with higher predicted probabilities of being at more advanced A/T PET status (c-index (95% CI) = 0.95 (0.93, 0.97), last column; Fig. 3b and Supplementary Table 9). More specifically, participants at CSF stages 0 and 1 (negative biomarkers and Aβ42/40 stages) had the highest probability of being A−T−, at CSF stages 2 and 3 of being A+T− and at CSF stages 4 and 5 of being A+T+. Only one participant was classified as A−T+ and was excluded from this analysis. As an additional analysis, we followed a similar approach with the recently proposed PET staging from the Alzheimer’s Association revised clinical guidelines (https://aaic.alz.org/diagnostic-criteria.asp). Similarly, higher CSF stages were associated with more advanced PET-based stages although with slightly lower accuracy (c-index (95% CI) = 0.92 (0.90, 0.94); Supplementary Fig. 3).

Finally, we also aimed at investigating whether our staging model could be used as a diagnostic tool (Fig. 3c,d). In the first analysis, we used the CSF staging model for predicting cognitive stages within the AD continuum (that is, excluding non-AD). Higher CSF stages were associated with more advanced cognitive stages (c-index (95% CI) = 0.88 (0.86, 0.91), first column; Fig. 3c and Supplementary Table 6). The model predicted that participants at CSF stage 0 had the highest probability of being CU−; at CSF stages 1 and 2, participants were more probably CU+ (as assessed by CSF); at CSF stage 3, participants were more probably MCI+; and, finally, at CSF stages 4 and 5, participants were more probably ADD+. Additionally, we also performed an analysis looking at the clinical stages based on the National Institute on Aging-Alzheimer’s Association (NIA-AAA) guidelines from 2018 (merging all dementia stages into one owing to sample size issues)^43^. Here, we also had a good predictive accuracy (c-index (95% CI) = 0.87 (0.84,0.89)), and we observed the expected pattern, with participants with subjective cognitive decline (SCD) mainly included in CSF stages 1–3 (Supplementary Fig. 4). Lastly, we aimed at differentiating cognitive impairment due to AD or due to other neurodegenerative diseases. We, therefore, compared patients with AD to patients with non-AD dementia, including only those with objective cognitive impairment (that is, patients with MCI and patients with dementia). Participants at CSF stage 2 or higher with objective cognitive impairment had a high probability of having AD as the cause of their cognitive impairment (AUC (95% CI) = 0.95 (0.93, 0.98), sensitivity = 0.97 and specificity = 0.75, last column; Fig. 3c,d and Supplementary Table 6).

### Assessment of longitudinal rates of change of AD biomarkers

Next, we used longitudinal imaging and cognitive data to assess how AD biomarkers change over time based on the baseline CSF stage classification (Supplementary Table 10). The rate of accumulation of Aβ aggregates as measured with PET (*n* = 218) increased at early CSF stages, reaching the highest values at CSF stage 2, and, thereafter, the rate decreased but still remained positive (Fig. 4 and Supplementary Table 11). On the other hand, the tau-PET (*n* = 312), cortical thickness (*n* = 300) and mPACC (*n* = 342) exhibited monotonic increases in rates of change over time, with the rates starting to be significantly different from contiguous CSF stages at CSF stage 3 (Fig. 4). Figure 4b depicts that tau-PET, followed by mPACC, had the highest rate of change (z-scored), whereas amyloid-PET and cortical thickness had lower rates of change that were in a similar range.

### Prediction of clinical progression

**Fig. 5:**
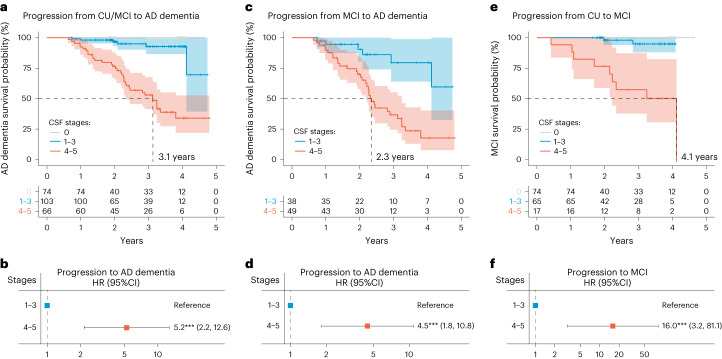
CSF stages for predicting clinical progression. Higher CSF stages groups (4–5) show higher HR of clinical progression compared to lower positive stages (1–3). Progression from CU or MCI at baseline to AD dementia is shown in **a** and **b**. Progression from MCI at baseline to AD dementia is shown in **c** and **d**. Progression from CU at baseline to MCI is shown in **e** and **f**. Kaplan–Meier curves (shaded area: 95% CI) as well as the number of participants per group and timepoint are shown in **a**, **c** and **e**, respectively. Cox proportional hazards models were used to calculate HR (95% CI) (square and error bars, respectively) of higher CSF stages (4–5) compared to the reference (1–3; **b**, **d** and **f**). These analyses were adjusted for age and sex in all cases and, additionally, for clinical status at baseline (CU or MCI) if appropriate. Dashed lines in **a**, **c** and **e** indicate the timepoint at which 50% of a group had progressed. Exact *P* values shown in the figure are as follows: **b**: *P* = 0.00025; **d**: *P* = 0.00097; **f**: *P* = 0.00082.

**Extended Data Fig. 6:**
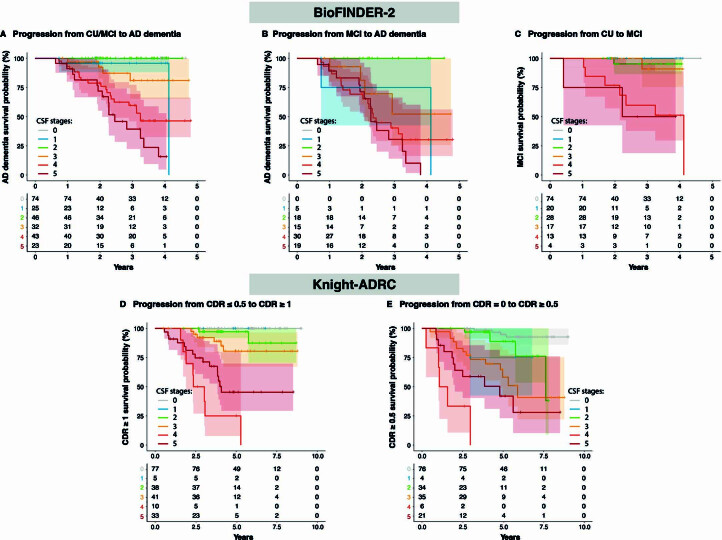
Individual CSF stages for predicting clinical progression. Kaplan-Meier curves (shaded area: 95%CI) for all individual CSF stages in BioFINDER-2 (A-C) and Knight-ADRC (D-E) participants. For BioFINDER-2, progression from CU or MCI at baseline to AD dementia is shown in A; progression from MCI at baseline to AD dementia is shown in B and; progression from CU at baseline to MCI is shown in C. For Knight-ADRC, progression from CDR = 0 or CDR = 0.5 at baseline to CDR≥1 is shown in D and; progression from CDR = 0 at baseline to CDR≥0.5 is shown in E. Abbreviations: AD, Alzheimer’s disease; CDR, clinical dementia rating; CSF, cerebrospinal fluid; CU, cognitively unimpaired; MCI, mild cognitive impairment.

In the next set of analyses, we tested whether our CSF staging model was useful for predicting subsequent clinical progression (up to 5 years of follow-up after the baseline visit). First, we tested the ability of our model to predict progression to AD dementia from CU or MCI status at baseline (progressors: *n* = 41). Based on Kaplan–Meier curves and Cox proportional hazards analyses (Fig. 5a), participants at higher CSF stages (4–5) at baseline had higher probability to progress to AD dementia than those at positive lower CSF stages (that is, 1–3), with 50% of these participants progressing at 3.1 years. When adjusting for age, sex and clinical status at baseline (that is, CU or MCI), the hazard ratio (HR) was 5.2 (95% CI: 2.2, 12.6, *P* < 0.001), when comparing participants at CSF stages 4 or 5 to participants at lower, but positive, CSF stages (1–3; Fig. 5b and Supplementary Table 12). When including only those with MCI at baseline (progressors: 38/88), we still found that those at CSF stages 4 or 5 at baseline had a significantly higher probability to progress to AD dementia (HR (95% CI) = 4.5 (1.8, 10.8), *P* < 0.001; Fig. 5c,d and Supplementary Table 12). After 2.3 years, half of these participants already progressed to AD dementia. Finally, we investigated the utility of the CSF staging model when predicting progression from CU to MCI status (progressors: 11/159). Again, those CU participants at higher CSF stages (4–5) at baseline were much more prone to progress to MCI with an HR of 16.0 (95% CI: 3.2, 81.1, *P* < 0.001; Fig. 5e,f and Supplementary Table 12) compared to those in stage 1–3, and 50% already progressed to MCI after 4.1 years, supporting the clinical utility of the proposed staging model. There were no progressors from CSF stage 0 in any case, which prevented us from comparing these participants with the other CSF stages groups. Kaplan–Meier curves for each individual CSF stage are depicted in Extended Data Fig. 6.

### Replication in an independent cohort

**Fig. 6:**
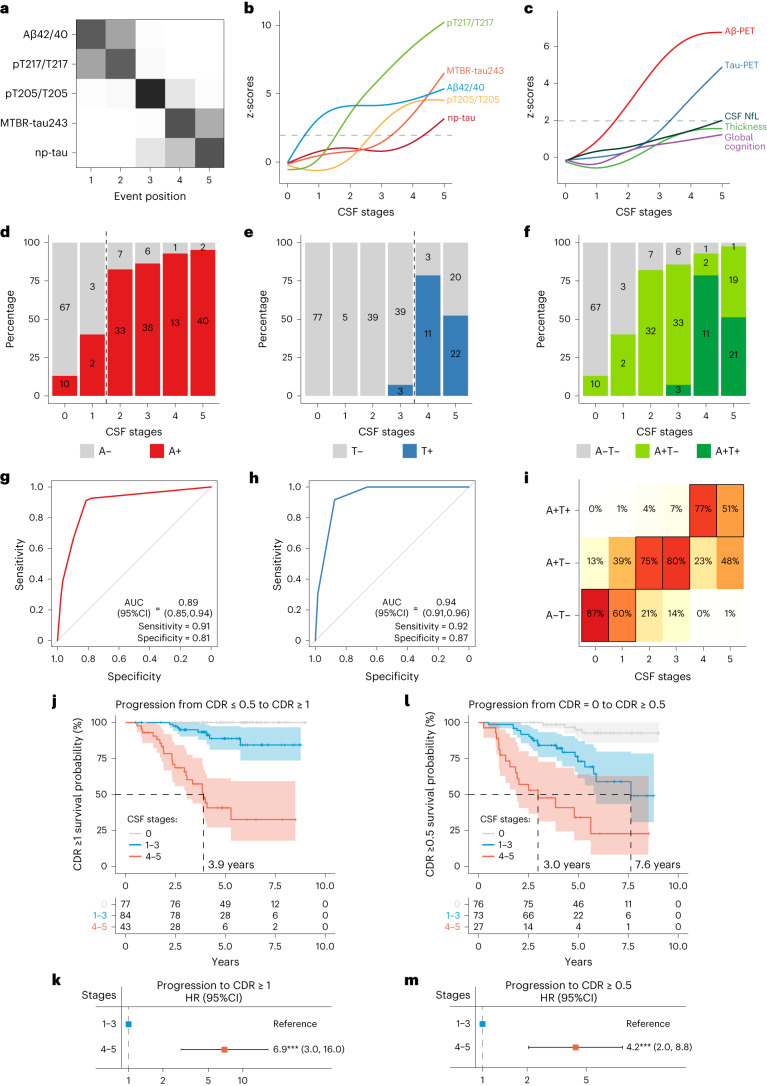
Replication of main analyses in Knight ADRC participants. Cross-validated confusion matrix of the CSF biomarkers of the model is shown in **a**. Darkness represents the probability of that biomarker becoming abnormal at that position, with black being 100%. Description of the CSF levels of the biomarkers included in the model by CSF stages are shown in **b**. Depiction of individual biomarker levels, not used in the creation of the model by CSF stages, are shown in **c**. All increases represent increase in abnormality. The horizontal line is drawn at z-score = 1.96, which represents 95% CI of the reference group (CU−). CSF stage 0 represents being classified as normal by the model. Prediction of amyloid-PET (**d**–**g**), tau-PET (**e**–**h**) and A/T status (by PET, **f**–**i**) are shown next. The number of participants in each category is colored in **d**–**f**. Numbers of participants in each category per CSF stage are shown within the bar plots. ROC curves were used to determine the CSF stage to optimally classify participants into positive/negative in each case (**g** and **h**). The optimal cutoff in each case is shown as a vertical dashed line in **d** and **e**, respectively. The heat map represents the predicted percentage of participants in each A/T group per CSF stage (**i**). The most probable (highest percentage) group per CSF stage is framed in black. Progression from CDR = 0 or CDR = 0.5 at baseline to CDR ≥ 1 is shown in **j** and **k** and from CDR = 0 to CDR ≥ 0.5 in **l** and **m**. Kaplan–Meier curves (shaded area: 95% CI) as well as the number of participants per group and timepoint are shown in **j** and **l**. Dashed lines indicate the timepoint at which 50% of a group had progressed. Cox proportional hazards models were used to calculate HR (95% CI) (square and error bars, respectively) of higher CSF stages (4–5) compared to the reference (1–3, **k** and **m**). Exact *P* values shown in the figure are as follows: **k**: *P* = 6.2 × 10^−6^; **m**: *P* = 0.00010.

**Extended Data Fig. 7:**
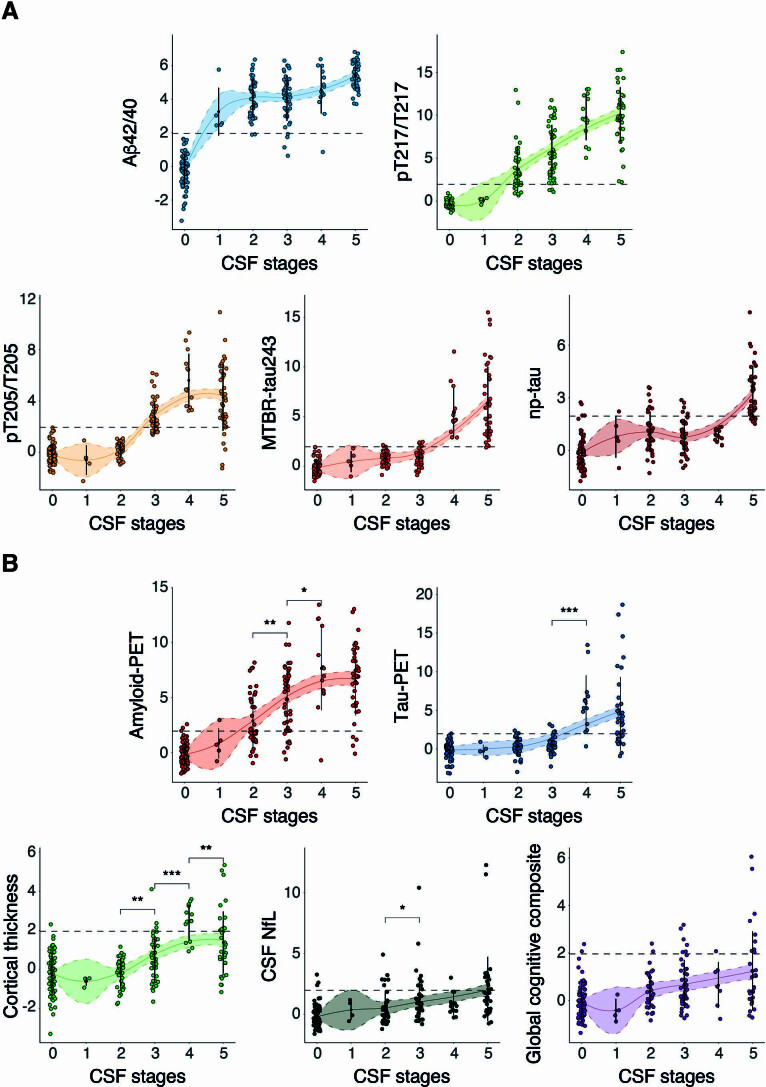
Individual biomarker levels by CSF stage in Knight-ADRC participants. Individual CSF biomarker levels, included in the model, by CSF stage participants are shown in A including all Knight-ADRC participants. Depiction of individual AD-biomarker levels, not used in the creation of the model, per CSF stage are shown in B. All biomarker levels are z-scored based on a group of CU- participants (n = 71) and all increases represent increase in abnormality. Significant differences in contiguous CSF stages are shown with asterisks (two-sided, FDR-corrected). Horizontal line is drawn at z-score = 1.96 which represents 95%CI of the reference group (CU-). Black central dot and vertical lines represent the mean and two standard deviations of each stage, respectively. Colored lines and bands represent the LOESS regression and its 95%CI. CSF stage 0 represent being classified as normal by the model. Black dots and vertical lines represent mean and SD per CSF stage. *: p < 0.05; **: p < 0.01; ***: p < 0.001. Exact p-values shown in the figure are, Amyloid-PET: 2-3: p = 0.003; 3-4: p = 0.005; Tau-PET: 3-4: p = 4.4·10^−10^; Cortical thickness: 2-3: p = 0.007; 3-4: p = 3.0·10^−5^; 4-5: p = 0.032; CSF NfL: 2-3: p = 0.35. Abbreviations: Aβ, amyloid-β; CI, confidence interval; CU-, cognitively unimpaired amyloid negative; CSF, cerebrospinal fluid; MMSE, Mini-Mental state examination; MTBR, microtubule binding region; NfL, neurofilament light; PET, positron emission tomography; np-tau, non-phosphorylated mid-region tau; pT, phosphorylated tau; SuStaIn, subtype and stage inference.

Finally, we replicated the staging model and the main analyses in the Knight ADRC cohort (*n* = 222; Table 1). SuStaIn selected one unique subtype as the optimal model with the same CSF abnormality ordering as the one previously obtained in BioFINDER-2 (Fig. 6a). In this cohort, however, there was slightly higher uncertainty between the ordering of the first two (Aβ42/40 and pT217/T217) and the last two (MTBR-tau243 and np-tau) stages. These differences may be due mostly to the difference in sample size, especially in more advanced AD cases (only nine mild AD dementia cases). Nonetheless, the overall behavior of these CSF biomarkers by the biomarker stages was similar to that in the main cohort (Fig. 6b and Supplementary Table 4). Furthermore, the other AD biomarkers available (not included in the CSF staging model) showed similar trajectories to those in the main sample (Fig. 6c and Supplementary Table 6). The main difference compared to BioFINDER-2 was the lower degree of abnormality for all markers in the last CSF stages. This might be explained by the lower number of advanced patient cases in this cohort. The individual plots for each CSF and imaging biomarker by CSF stages are shown in Extended Data Fig. 7. Details of participant characteristics (Extended Data Fig. 2), tau-PET binding in different regions (Extended Data Fig. 4 and Supplementary Table 7) and other cognitive measures (Extended Data Fig. 5 and Supplementary Table 8) per CSF stage can be found in the Extended Data. Stability analyses, within participants with available longitudinal CSF measures (*n* = 51; Supplementary Table 13), also showed that most participants remained at the same stage (*n* = 46, 90.2%) or progressed (*n* = 4, 7.8%) at follow-up (Extended Data Fig. 3c,d).

We also calculated the optimal CSF stages for predicting Aβ-PET and tau-PET positivity using ROC curves. As in the case of BioFINDER-2, CSF stage 2 was optimal for predicting amyloid-PET positivity (AUC (95% CI) = 0.89 (0.85, 0.94); Fig. 6d,g and Supplementary Table 9), whereas CSF stage 4 was optimal for predicting tau-PET positivity (AUC (95% CI) = 0.94 (0.91, 0.96); Fig. 6e,h). Consistent with findings in BioFINDER-2, higher CSF stages were predictive of more advanced A/T stages, as assessed by PET (c-index (95% CI) = 0.89 (0.86, 0.92); Fig. 6f,i and Supplementary Table 9). Being at CSF stages 0 and 1 was highly predictive of being A−T−; being at CSF stages 2 and 3 was predictive of being A+T−; and being at CSF stages 4 and 5 was predictive of being A+T+.

Finally, we investigated the prognostic capacity of our model for predicting progression to Clinical Dementia Rating (CDR) ≥ 1 (AD dementia, progressors: 41/218) and CDR ≥ 0.5 (MCI or very mild AD dementia, progressors: 30/214). We found that CU (CDR = 0) and very mild AD (CDR = 0.5) participants at the highest CSF stages (4–5) exhibited an increased risk (HR (95% CI) = 6.9 (3.0, 16.0), *P* < 0.001) of progressing to AD dementia (CDR ≥ 1) at follow-up, even when adjusting for age, sex and clinical status (that is, CDR = 0 or CDR = 0.5) at baseline, compared to participants at CSF stages 1–3 (Fig. 6j,k and Supplementary Table 12). Half of this group already progressed to CDR ≥ 1 after 3.9 years. Similarly, CU participants at higher CSF stages (that is, 4–5) had higher risk (HR (95% CI) = 4.2 (2.0, 8.8), *P* < 0.001) of progressing to very mild AD or more advanced disease stages when compared to participants at lower, but positive, CSF stages (1–3; Fig. 6l,m and Supplementary Table 12), with 50% of them progressing after 3.0 years, whereas, for the 1–3 group, it took 7.6 years. In this case, participants at CSF stages 1–3 also showed significant higher risk to progress to CDR ≥ 0.5 than those at CSF stage 0 (HR (95% CI) = 5.0 (1.6, 15.0), *P* = 0.005). There were no progressors to CDR ≥ 1 at CSF stage 0, which prevented us from comparing this group to the others. Kaplan–Meier curves for each individual CSF stage are depicted in Extended Data Fig. 6.

## Discussion

**Fig. 7:**
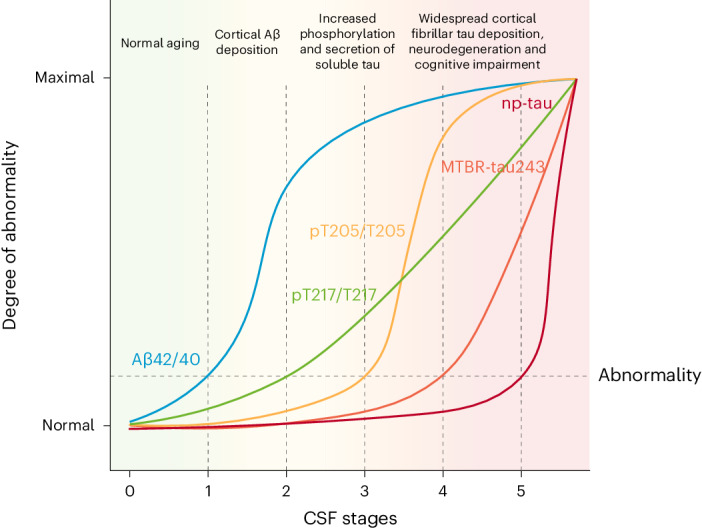
CSF stages and disease progression. Simplified version of the CSF biomarkers trajectory across CSF s nship with disease progression. The text above is hypothetical and is based on previous studies^4,28,29,32,53,86^.

In this study, we created and evaluated a staging model for AD using five CSF biomarkers reflecting abnormalities of soluble Aβ and different soluble tau species (Fig. 7). We demonstrate here that a single CSF collection is sufficient to accurately stage participants representing the entire AD continuum. This is possible because CSF biomarker abnormalities followed a stereotypical trajectory in all participants, which enabled a single staging model usable for everyone. Notably, we were able to relate the CSF stages of our model to abnormality in other well-described AD biomarkers, such as amyloid-PET and tau-PET, in magnetic resonance imaging (MRI) and in cognitive measures. Furthermore, our CSF staging model was able to accurately predict positivity of the imaging biomarkers of Aβ and tau and to predict A/T status, as assessed by PET. The CSF staging model was also related to cognitive stages and was able to differentiate cognitive impairment due to AD from other dementias. Notably, we also observed different longitudinal rates of change of AD biomarkers at different CSF stages, which may allow us to determine which participants will progress more in key aspects of the disease. In addition, we showed that participants in the more advanced stages of our CSF-based model were at higher risk for clinical decline. Finally, we were able to replicate the model and main results in an independent cohort. Altogether, these results support the validity of our CSF staging model and indicate promising clinical utility, suggesting that it may be useful in clinical practice and in clinical trials if further validated^44,45^.

The first aim of this analysis was to establish whether there was a stereotypic ordering in when key CSF biomarkers become abnormal. SuStaIn is an optimal approach to answer this question as it allows the modeling of different trajectories, if existent for subgroups of the whole sample, using cross-sectional data^41^, as has been successfully applied to imaging biomarkers^35,36,46^. We observed that the CSF biomarkers investigated in this study became abnormal in a particular sequence and, more importantly, that this sequence did not vary systematically across participants. This result is important by itself as it tells us that there may be a single cascade of events that leads to sequential abnormality of these soluble proteins in the brain, common to all patients with AD. Previous studies already suggested that changes in the levels of tau fragments phosphorylated at different sites may be linked mechanistically and could be associated with disease stages^47–51^. Based on our results, Aβ plaques reflected by an imbalance of soluble amyloid species (that is, low Aβ42/40) may drive hyper-phosphorylation of tau in early phosphorylation site (pT217/T217), as previously suggested by human and animal data^52,53^, which would subsequently be followed by hyper-phosphorylation in later site (pT205/T205) and eventually increase other tau fragments (MTBR-tau243 and np-tau) due to tangles formation and neurodegeneration. Notably, this sequence of events is in line with previous literature^54,55^ and demonstrates that late-onset sporadic AD molecular pathway matches the same sequence of events as autosomal dominant AD^29^. Exploring in detail this cascade of events may provide mechanistic insights into disease pathology and progression. In turn, it could have important consequences in drug development, as targeting some of the earliest events of this sequence may stop or reduce subsequent events in the cascade and, thereby, have a significant effect on tau aggregation^47,51,56^.

Perhaps the most important result of our study was proving the utility of a CSF model as a method to stage AD in vivo^44,45^. In our model, CSF stages could be related to main molecular changes and clinical tipping points in the course of the disease, including abnormal levels of deposited Aβ (CSF stage 2: pT217/T217)^19,20,23,25,26,28,29,32^ and tau (CSF stage 3: pT205/T205) (refs. ^28,29,32^), early cognitive impairment (CSF stage 4: MTBR-tau243) (ref. ^32^) and neurodegeneration (CSF stage 5: np-tau), following the expected pattern. With the objective of characterizing the molecular status of the participants using our model, we observed that participants at CSF stages 2 and 3 (pT217/T217 and pT205/T205 stages) could be categorized with high accuracy as being Aβ positive and tau negative by PET (A+T−), whereas participants at CSF stage 4 (MTBR-tau243) or higher were amyloid-PET and tau-PET positive (A+T+)^57^. Notably, these cutpoints were reproduced in the Knight ADRC cohort, even using different PET tracers and quantification methods, supporting the consistency of the model. Being able to accurately assess Aβ and tau status with a single CSF collection may be very useful to select the optimal participants for a clinical trial, such as has been done in the donanemab trial (NCT03367403)^11^, without the need of acquiring both an amyloid-PET and a tau-PET scan to determine if a patient is eligible for treatment. In BioFINDER-2, we also observed the diagnostic utility of this CSF staging model, as it was able to accurately discern AD-related from non-AD-related cognitive impairment and could differentiate cognitive and clinical stages. Thus, the use of our model as a diagnostic tool may have important consequences at the clinical level as well.

Notably, our CSF staging model also showed prognostic utility. First, we observed that participants at different CSF stages showed different rates of change in multiple biomarkers. For instance, rates of Aβ accumulation across CSF stages showed the previously reported inverted U shape^3,58^, with participants at CSF stage 2 (pT217/T217) exhibiting the highest rates of change. On the other hand, the other imaging biomarkers and cognitive scores showed increased rate of change with increasing CSF stages, only plateauing at the last stage, as expected^59^. These results support the use of our staging model as an enrichment technique for clinical trials^60^. But, more importantly, we also observed that the CSF staging model was able to predict clinical progression. Being at the later stages of our model increased the risk of progressing to AD dementia, even when accounting for cognitive status at baseline (Fig. 5). Furthermore, we also observed a higher risk of progressing to MCI or very mild AD, although this analysis should be replicated in larger cohorts with longer follow-up. Notably, the prognostic ability of our CSF staging model was replicated in the Knight ADRC cohort. These results suggest a clear prognostic utility on staging participants based on their CSF profile, which may imply substantial reductions in costs and complexity compared to previous staging methods based on PET^34,36,37,61^.

We view the present model as a first step toward providing meaningful disease progression staging using a single CSF measurement^44,45^. We expect that additional biomarkers will be included to the model either to gain further granularity in specific disease stages or to signify to other pathophysiological events (for example, microglial reactivity)^62^. Being able to measure several pathophysiological abnormalities using one sample is one of the main advantages of using fluid samples instead of PET for staging. Another advantage of this model is that the financial and infrastructure cost of CSF is low compared to other measures, such as PET. Looking toward the future, we hope to be able translate these results into plasma biomarkers, which would facilitate even greater availability and cost-effectiveness. Widespread use of our fluid biomarker staging model in primary care would likely require replacing CSF measures with plasma measures without greatly sacrificing model performance. Efforts in this direction are currently underway, but development of reliable plasma assays for pT205/T205 and MTBR-tau243 is still ongoing.

The main strength of this study is the proven utility of the model, which was replicated in an independent cohort and, thereby, supports the generalizability of our staging model. Another important strength is the use of several biomarkers measured with very high-performing assays^28,32,63^, which is crucial for the accurate assessment of pathology^4^. However, some limitations must be recognized. Although we included CSF biomarkers with proven utility, we acknowledge that there are some other interesting markers, such as p-tau235 (ref. ^64^), that have not been analyzed in this study. However, we think that our CSF staging model in its current form was still successful at signaling the main inflection points of the disease. Furthermore, p-tau231, which is thought to become abnormal early in the disease^20–22^, although not always^25,63^, was excluded from our model as it followed a similar abnormality tendency as pT217/T217, without providing better performance for staging than the latter. We hypothesize that this may be in part related to difference in analytical performances, as the mass spectrometry platform used in our study provided rather higher coefficient of variation for pT231/T231 measurements (12–18% compared to 5–7% for pT217/T217). Future studies in earlier cohorts or with optimized assays for measuring p-tau231 should test whether the present model could be improved. Another important issue is that we acknowledge that CSF collection requires trained clinicians, and we plan to move toward a plasma-based staging model when these biomarkers become available. A replication of these results in a more diverse population is also needed to confirm the utility of our model in a less selected population. Furthermore, we could not test the effects that other comorbid pathologies may have on our staging system. This should be explored in future studies with available neuropathological information. We would also like to point out that the CSF stages proposed here are related to events of the disease and not to time. Thus, it may be possible that the time for progressing from one CSF stage to the next varies markedly depending on the CSF stage at baseline. We acknowledge that the combined use of the continuous measures of the selected biomarkers could render similar accuracies to those obtained by the CSF stages for some predictive purposes. Nonetheless, we think that the simplicity of our model is also a key point for its future utility in clinical practice. Finally, we cannot rule out that the staging of our model can be affected by biomarker sensitivity, such that more sensitive biomarkers may be more likely to be ordered earlier in the model. This has no bearing on the predictive value of the model as described here, and the biomarker ordering that we discovered both conforms with previous knowledge of AD biomarker sequencing and predicts other biomarker changes in a manner coherent with expectations. Nonetheless, it will be important for future models to make use of the most sensitive biomarkers available and, in doing so, ensure that they are calibrated to these new data.

In conclusion, in the present study, we developed an accurate staging model for AD based on only five CSF biomarkers, and we evaluated it in two large independent cohorts. We showed that the model is stable and accurately reflects biomarker changes in AD, providing an easier and cheaper method for characterization of participants for both clinical setting and trials. Furthermore, our model has demonstrated its utility for prognosis, being able to identify participants with more pronounced longitudinal changes in AD biomarkers as well as those individuals with higher risk of deteriorating in cognitive status. This CSF staging model may be a useful, cheap and accessible method in clinical trials for optimal selection of study participants and as a surrogate outcome measure. Furthermore, the staging model has great potential for use in clinical practice in the diagnostic and prognostic workup of patients with cognitive symptoms and potentially also for selecting optimal candidates for disease-modifying treatments. In addition, we expect that it may have an influence on the update of the A/T/(N) criteria^57^. Altogether, our staging model may be an important step toward a more sophisticated personalized medicine approach of AD, which will be key with the advancement of disease-modifying treatments.

## Methods

### Participants

#### BioFINDER-2

We assessed 426 participants from the Swedish BioFINDER-2 study (NCT03174938)^19^, with the complete set of CSF biomarkers available. Participants were recruited at Skåne University Hospital and the Hospital of Ängelholm in Sweden. These participants also had amyloid-PET (*n* = 251), tau-PET (*n* = 417), MRI (*n* = 420) and cognitive assessment (*n* = 426). In addition, 220 participants had available CSF biomarkers at follow-up (mean time (s.d.) = 2.05 (0.22) years). Inclusion and exclusion criterion for this study were detailed previously^19^. In summary, CU participants do not fulfil criteria for MCI or dementia according to the *Diagnostic and Statistical Manual of Mental Disorders*, Fifth Edition (DSM-5)^65^. Participants with SCD were considered as CU, in accordance with the research framework by the NIA-AA^43^. Participants with MCI had a Mini-Mental State Examination (MMSE) score above 23; they did not fulfil the criteria for major neurocognitive disorder (dementia) according to DSM-5; and they performed worse than −1.5 s.d. in at least one cognitive domain according to age and education-stratified test norms. AD dementia was diagnosed according to the DSM-5 criteria for major neurocognitive disorder due to AD, and an abnormal biomarker for Aβ pathology was also required. Participants fulfilling the criteria for any other dementia were categorized as non-AD dementia, as previously described^19^.

#### Knight ADRC

The Knight ADRC cohort consisted of community-dwelling volunteers enrolled in studies of memory and aging at Washington University in St. Louis. All Knight ADRC participants underwent a comprehensive clinical assessment that included a detailed interview of a collateral source, a neurological examination of the participant, the CDR^66^ and the MMSE^67^. Individuals with a CDR of 0.5 or higher were considered to have a dementia syndrome, and the probable etiology of the dementia syndrome was formulated by clinicians based on clinical features in accordance with standard criteria and methods^68^. In the Knight ADRC cohort, participants were categorized as CU if they scored CDR = 0, either Aβ negative or Aβ positive (CU− and CU+, respectively); patients with very mild AD if they scored CDR = 0.5; and patients with mild AD dementia if they scored CDR ≥ 1 and the clinical syndrome was typical of symptomatic AD. Participants with CDR ≥ 0.5 with different etiology were assessed as being patients with ‘Other dementia’ regardless of their amyloid status.

All participants gave written informed consent, and ethical approval was granted by the Regional Ethical Committee in Lund, Sweden (protocol: 2016_1053), and the Washington University Human Research Protection Office (protocol: 201109100).

### Fluid biomarkers

Measurement of CSF tau species (that is, pT205/T205, pT217/T217, pT231/T231, MTBR-tau243 and np-tau variants) was performed at Washington University in both cohorts using the developed IP/MS method, as previously detailed^32^. In brief, Tau1 (generated by Nicholas Kanaan) and HJ series (HJ8.5, HJ8.7, HJ32.11 and HJ34.8) antibodies (generated by David Holtzman) were used. In BioFINDER-2, CSF levels of Aβ42/40 and NfL were measured using the Elecsys platform, as previously described^19^. Aβ positivity was assessed using CSF Aβ42/40 (<0.080), unless otherwise stated, based on Gaussian mixture modeling. In Knight ADRC, CSF Aβ42/40 levels were measured as described previously^28,69^. The CSF Aβ42/40 positivity threshold (0.0673) had the maximum combined sensitivity and specificity in distinguishing amyloid-PET status. CSF NfL was measured with a commercial ELISA kit (UMAN Diagnostics), as described previously^70^. Data analysis was performed blinded to the diagnostics of the participants.

### Image acquisition and processing

Image acquisition and processing details from BioFINDER-2 were previously reported^19^. In brief, amyloid-PET and tau-PET were acquired using [^18^F]flutemetamol and [^18^F]RO948, respectively. Amyloid-PET binding was measured as standardized uptake value ratio (SUVR) using a neocortical meta-ROI and with the cerebellar gray as a reference region. Of note, most of the patients with AD dementia did not undergo amyloid-PET in BioFINDER-2 owing to the study design. For main analyses, tau-PET binding was measured in a temporal meta-ROI^71^, which included entorhinal, amygdala, parahippocampal, fusiform, inferior temporal and middle temporal ROIs, using the inferior cerebellar cortex as a reference region without partial volume correction. Additionally, tau-PET binding was also measured in regions covering early (Braak I), intermediate (Braak III–IV) and late (Braak V–VI) tau deposition areas^42^. For assessing cortical thickness, T1-weighted anatomical magnetization-prepared rapid gradient echo (MPRAGE) images (1-mm isotropic voxels) were used. A cortical thickness meta-ROI was calculated including entorhinal, inferior temporal, middle temporal and fusiform using FreeSurfer (version 6.0; https://surfer.nmr.mgh.harvard.edu) parcellation, which are areas known to be susceptible to AD-related atrophy^72^.

Methodological details for imaging processing and quantification for the Knight ADRC cohort were also previously reported^72,73^. In brief, MPRAGE data were processed using FreeSurfer (version 6.0) to generate ROIs. Amyloid-PET was acquired with either [^11^C]PIB or [^18^F]florbetapir and was quantified in a neocortical meta-ROI using cerebellar gray as a reference region. SUVR values were transformed to Centiloids^74^ to allow direct comparison between tracers using previously validated transformations^75^. [^18^F]flortaucipir ([^18^F]AV1451) was used as a tau-PET tracer, and images were quantified in the same temporal meta-ROI as in BioFINDER-2 and assessed as positive if SUVR > 1.32, based on previous work^76^. The same additional regions as in BioFINDER-2 were also used to quantify tau-PET binding in early, intermediate and late tau deposition regions. In all cases, cerebellar gray was used as a reference region. T1-weighted images were used to measure cortical thickness using the same approach as in the BioFINDER-2 cohort.

### Neuropsychological testing

mPACC and a global cognitive composite were used as the main cognitive outcome in BioFINDER-2 and Knight ADRC participants, respectively. In BioFINDER-2 participants, the mPACC-5 composite was calculated using mean of z-scores of Alzheimer’s Disease Assessment Scale (ADAS) delayed recall (weighted double), animal fluency, MMSE^67^ and Trail Making Test-A (TMT-A)^77^, as a sensitive measure of early cognitive impairment^78^. We calculated z-scores with a group of CU− as reference. Furthermore, we also calculated several cognitive composites by averaging z-scores of different cognitive tests. For the memory composite, we used ADAS delayed and immediate word recall; for the executive function composite, we used TMT-A, TMT-B and the symbol digit test; for the language composite, we used the animal fluency test and the Boston Naming Test (BNT) total score^79^; and, finally, for the visuo-spatial composite, we used the visual object and space perception (VOSP) cube and letters tests.

In Knight ADRC, the global cognitive composite was created using mean of z-scores of free and cued selective reminding test (FCSRT) free recall^80^, animal fluency, TMT-A and TMT-B. We calculated z-scores from a CU− group as a reference. For the executive function composite, we used TMT-A and TMT-B. We could not obtain any other cognitive composite similar to those derived in BioFINDER-2 due to lack of similar tests. However, we selected individual tests to try to recapitulate similar cognitive measures. For memory, we used FCSRT, and, for language, we used animal fluency. No tests were available related to visuo-spatial capacity.

### Model creation

Model development was done with SuStaIn^41^ using cross-sectional data of amyloid-positive participants based on CSF Aβ42/40 levels. We selected these participants because we wanted to create a staging model focused on AD. However, using the whole sample also rendered a single subtype model with the same ordering in the abnormality of the CSF biomarkers. SuStaIn is a Bayesian technique that unravels temporal progression patterns (stages), allowing for multiple different trajectories (subtypes). For our purpose, we used the event-based model^81^ (or mixture SuStaIn^82^), in which the input data are the probability of each biomarker being abnormal for each participant. In our case, we used a Gaussian mixture modeling approach (with two Gaussians) to obtain these probabilities. With this information, SuStaIn provides the maximum likelihood sequence by which biomarkers become abnormal and gives a probability for this ordering, for all subtypes. The number of SuStaIn stages is defined by the number of biomarkers provided to the model (that is, one per biomarker plus a biomarker negative stage). The selection of the optimal number was determined using cross-validation, optimizing on cross-validation-based information criterion (CVIC), and out-of-sample log-likelihood was calculated. The optimal number of subtypes was then selected based on these criteria, using the minimal number of subtypes that had the lowest CVIC and higher log-likelihood^41^. In this study, we used pySuStaIn^83^, a Python implementation of the original method (downloaded in August 2022).

In our initial model with BioFINDER-2 participants, we included all biomarkers available and performed the cross-correlation in models with one, two and three subtypes. We included only one non-phosphorylated peptide due to the extremely high correlation that all have among them (*r* ≥ 0.98; Supplementary Fig. 5). Although CVIC measures were lower in the three-subtype model, the similar log-likelihood in all three models supported the one-subtype model as the optimal one due to its lower complexity^84^. Upon examining the outcome of this model, we observed that pT217/T217, pT231/T231 and pT181/T181 position certainty was low, as they seemed to compete for the second position (Extended Data Fig. 1a). To avoid stages with low certainty, we decided to try to optimize this model through iterative removal of these biomarkers. All the possible combinations were created (that is, removing pT217/T217 and/or pT231/T231 and/or pT181/T181) and compared using the CVIC (Extended Data Fig. 1b). We observed that models including only one of these biomarkers (models 5–7) were better than those including two (models 2–4) or all three (model 1). Furthermore, models including pT181/T181 performed worse, and those including pT217/T217 performed better. Thus, the optimal model was selected as that including only pT217/T217 (model 7). Once the biomarkers to be included in the model were selected, we repeated the cross-validation with models up to three subtypes. Comparing CVIC and log-likelihood values, we once again selected the one-subtype model as the optimal (Extended Data Fig. 1c). Based on this cross-validated model, we then staged all BioFINDER-2 participants.

We also did some sensitivity analyses in the creation of the model. First, we created a model including all np-tau variants available by the mass spectrometry analyses. We observed that the high correlation between the different np-tau variants prevented the algorithm from finding a clear ordering among them but not for the other included AD biomarkers (Supplementary Fig. 6). This supports the inclusion of a single np-tau in the final model. Next, we created new models using different np-tau fragments that rendered very similar results (Supplementary Methods). Furthermore, we also created the model in 10 different random samples (random shuffle per biomarker without resampling). With this sensitivity analysis, we showed that the performance of our final model on explaining the provided data was significantly better than what could be expected from a model created by chance. Finally, we simulated new datasets with two and three underlying subtypes to assess the minimal size a subtype must be to be detected by SuStaIn (Supplementary Methods). We observed that SuStaIn was able to detect two and three underlying subtypes until the smaller subtypes included more than 5% of the original sample (Supplementary Figs. 8 and 9). Thus, we showed that the lack of different subtypes in our main analysis is, with high probability, due to the existence of one single sequence and not due to sample size problems.

For Knight ADRC, we followed the same main approach. We first started with seven biomarkers and tested the optimal number of subtypes, which was, again, one (CVIC: subtype 1 = 629.5, subtype 2 = 649.4, subtype 3 = 658.4, log-likelihood: subtype 1 = −32.3, subtype 2 = −33.3, subtype 3 = −33.8; Supplementary Fig. 10a). Then, given the high overlap between biomarkers, we tested removing pT217/T217, pT231/T231 and/or pT181/T181 again. Based on CVIC (model 1: 629.5, model 2: 590.1, model 3: 564.6, model 4: 567.1, model 5: 520.7, model 6: 524.0 and model 7: 497.2) and log-likelihood (median: model 1: −31.8, model 2: −29.8, model 3: −28.1, model 4: −28.4, model 5: −25.9, model 6: −26.1 and model 7: −24.8) metrics, the model with only pT217/T217 (model 7) was again selected as the optimal (Supplementary Fig. 10b). Finally, we created models for 1–3 subtypes and based on CVIC (subtypes: 1 = 497.2, 2 = 524.5 and 3 = 550.6) and log-likelihood (median: 1 = −24.8, 2 = −27.0 and 3 = −28.3), and the less complex model (that is, one subtype) was selected as the best fit to the data (Supplementary Fig. 10c). We then staged all Knight ADRC participants based on this cross-validated model. As a sensitivity analysis, we also used the model created with BioFINDER-2 data and applied to the Knight ADRC cohort instead of creating the model in the Knight ADRC cohort. Very similar results were found, with 213 of 222 participants (96%) assigned to the same stage as when we fitted the model on the Knight ADRC data. Of note, all nine cases changed from CSF stage 5 (creating the model in Knight ADRC) to CSF stage 4 (using BioFINDER-2 model). This change may be due to the lower severity of Knight ADRC participants compared to those in BioFINDER-2, as they all had the lowest np-tau levels in CSF stage 5.

**Extended Data Fig. 8:**
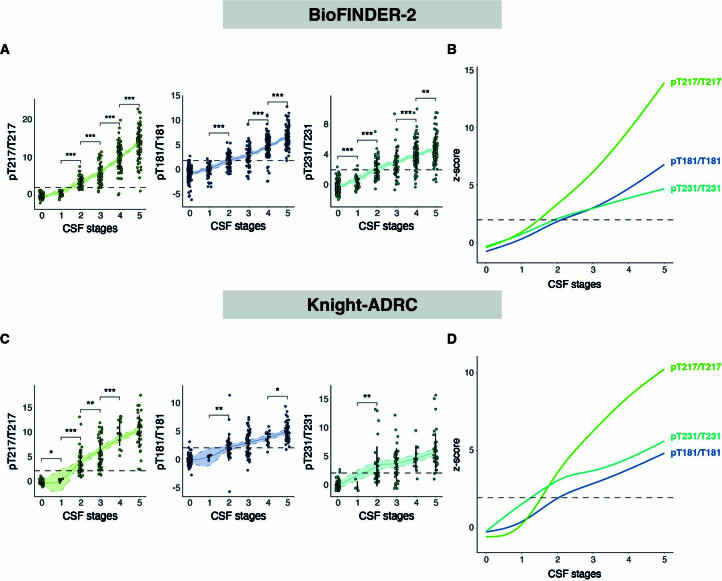
Excluded CSF biomarkers by CSF stage. Depiction of the CSF biomarkers excluded in the optimal model (pT231/T231 and pT181/T181) by CSF stage in BioFINDER-2 (A-B) and Knight-ADRC (C-D) participants. CSF pT217/T217 is also shown for comparison. CSF levels are z-scored based on a group of CU- participants (BioFINDER-2: n = 63, Knight-ADRC: n = 71) and all increases represent increase in abnormality. Significant differences in contiguous CSF stages are shown with asterisks (two-sided, FDR-corrected). Horizontal line is drawn at z-score = 1.96 which represents 95%CI of the reference group (CU-). Black central dot and vertical lines in A and C represent the mean and two standard deviations of each stage, respectively. Colored lines and bands represent the LOESS regression and its 95%CI. Smoothed LOESS lines of all CSF biomarkers are shown in B (BioFIDNER-2) and D (Knight-ADRC) for comparison. CSF stage 0 represent being classified as normal by the model. Black dots and vertical lines represent mean and SD per CSF stage, respectively. *: p < 0.05; **: p < 0.01; ***: p < 0.001. Exact p-values shown in the figure are, pT217/T217: 1-2: p = 3.7·10^−15^; 2-3: p = 3.1·10^−5^; 3-4: p = 3.3·10^−12^; 4-5: p = 4.7·10^−10^; pT181/T181: 1-2: p = 1.3·10^−7^; 3-4: p = 1.8·10^−7^; 4-5: p = 1.8·10^−7^pT231/T231: 0-1: p = 0.0004; 1-2: p = 2.9·10^−9^; 3-4: p = 9.8·10^−5^; 4-5: p = 0.007 for BioFINDER-2; and pT217/T217: 0-1: p = 0.041; 1-2: p = 0.0004; 2-3: p = 0.0008; 3-4: p = 3.7·10^−6^; pT181/T181: 1-2: p = 0.006; 4-5: p = 0.012; pT231/T231: 1-2: p = 0.0019 for Knight-ADRC. Abbreviations: Aβ, amyloid-β; CI, confidence interval; CU-, cognitively unimpaired amyloid negative; CSF, cerebrospinal fluid; LOESS, locally estimated scatterplot smoothing; MTBR, microtubule binding region; np-tau, non-phosphorylated mid-region tau; pT, phosphorylated tau; SuStaIn, subtype and stage inference.

As a sensitivity analysis, we compared the levels of the two biomarkers excluded (pT231/T231 and pT181/T181) with those of pT217/T217 in the optimal model. In summary, we observed that these biomarkers followed a similar trajectory across CSF stages as pT217/T217 although with lower increases in the two cohorts (Extended Data Fig. 8a,b), which supports our decision of removing them from the model to have more stable and independent stages.

### Statistics and reproducibility

All biomarkers were z-scored using participants older than 60 years from the CU− group as a reference (BioFINDER-2: *n* = 63 and Knight ADRC: *n* = 71). When necessary, biomarker data were inverted such that higher z-scores related to higher abnormality across all biomarkers. Differences in biomarkers by CSF stages were assessed using the Kruskal–Wallis test. The Wilcoxon test was used for post hoc comparisons adjusted for multiple comparisons with false discovery rate (FDR) correction (only differences in consecutive CSF stages are shown in the figures, but all comparisons are shown in supplementary tables). For categorical data (that is, sex, *APOE* carriership and diagnosis), we used chi-squared tests. LOESS regressions were used to fit the progression of biomarker abnormalities across the CSF stages. ROC curves were used to assess the utility of CSF stages for predicting amyloid-PET and tau-PET positivity and to compare AD to non-AD objective cognitive impairment (MCI or dementia states). Maximization of Youden’s index was used to select the optimal CSF stage cutoff in each case (‘pROC’ and ‘cutpointr’ packages were used). For ordinal categories (that is, A/T PET status and diagnosis), ordinal logistic regression models were used (‘MASS’ and ‘lmr’ packages). An equivalent measure to AUC, the c-index, was used to assess the performance of the CSF staging^85^. CIs were calculated using bootstrapping. Predicted probabilities of the outcome groups per each CSF stages were calculated using the ‘predict’ function. For longitudinal analyses, we first calculated longitudinal rates of change using linear regression models individually for each participant and biomarker. For each biomarker, we compared participants’ rates of change by their CSF stages at baseline as done in the cross-sectional analyses. LOESS regressions were also used to fit the progression of biomarkers’ rates of change across the CSF stages. One participant with a very negative rate of change in amyloid-PET (z-score < −1.8) was considered an outlier by visual inspection and was excluded from the analysis. Kaplan–Meier curves were used to assess clinical progression using the ‘survival’ and ‘survminer’ packages. Cox proportional hazards models were used to calculate the risk of clinical progression adjusting for age and sex in all cases and further baseline clinical status if necessary.

All analyses were performed with R (version 4.1.0). Two-sided *P* values less than 0.05 were considered statistically significant. For comparisons between CSF stages (that is, biomarker levels and rates of change), FDR correction was applied to account for multiple comparisons. All plots were done with the ‘ggplot’ package. Data distribution was assumed to be normal, but this was not formally tested. No statistical methods were used to pre-determine sample sizes, but our sample sizes per number of biomarkers are similar to those reported in previous publications^36,41^. Data collection was performed blinded to diagnostic characteristics.

### Reporting summary

Further information on research design is available in the Nature Portfolio Reporting Summary linked to this article.

### Supplementary information

Supplementary Information Supplementary Methods: Sensitivity analyses on model creation. Supplementary Data: Supplementary Table 1: Demographic characteristics by diagnosis. Supplementary Table 2: Vascular information from BioFINDER-2 participants. Supplementary Table 3: Diagnosis description of non-AD patients in the BioFINDER-2 cohort. Supplementary Table 4: Characteristics of BioFINDER-2 participants with follow-up CSF data. Supplementary Table 5: Statistics of each CSF biomarker and their differences by CSF stage. Supplementary Fig. 1: Description of CSF staging model by vascular risk factors. Supplementary Fig. 2: Description of CSF staging model by vascular pathologies observed in MRI. Supplementary Table 6: Comparison of BioFINDER-2 participants with and without follow-up CSF data. Supplementary Table 7: Statistics of AD biomarkers and their differences by CSF stage. Supplementary Table 8: Statistics of tau-PET binding in different regions and their differences by CSF stage. Supplementary Table 9: Statistics of cognitive composites and their differences by CSF stage. Supplementary Table 10: CSF stages for predicting predicting A/T status and as a diagnostic tool. Supplementary Fig. 3: CSF stages for predicting PET stages. Supplementary Fig. 4: CSF stages for predicting clinical stages. Supplementary Table 11: Characteristics of BioFINDER-2 participants with follow-up AD biomarkers. Supplementary Table 12: Statistics of AD biomarkers longitudinal rates of change and their differences by CSF stage. Supplementary Table 13: CSF stages for predicting disease progression. Supplementary Table 14: Characteristics of Knight ADRC participants with follow-up CSF data. Supplementary Fig. 5: Cross-correlation among biomarkers. Supplementary Fig. 6: Confusion matrix of the ordering of the model when all np-tau fragments are included. Supplementary Fig. 7: Creation of the model in 10 random samples. Supplementary Fig. 8: Simulation of two-subtype models with decreasing prevalence. Supplementary Fig. 9: Simulation of three-subtype models with decreasing prevalence. Supplementary Fig. 10: Creation and optimization of the model in the Knight ADRC cohort.

Reporting Summary

## Data Availability

The datasets generated and/or analyzed during the present study are available from the authors (O.H and R.J.B). The corresponding author will share datasets within the restrictions of institutional review board ethics approvals upon reasonable request. For BioFINDER-2 data, anonymized data will be shared by request from a qualified academic investigator for the sole purpose of replicating procedures and results presented in this article and as long as data transfer is in agreement with European Union legislation on the general data protection regulation and decisions by the Ethical Review Board of Sweden and Region Skåne, which should be regulated in a material transfer agreement. Knight ADRC data are available to qualified investigators who have a proposal approved by an institutional committee (https://knightadrc.wustl.edu/Research/ResourceRequest.htm). The study must be approved by an institutional review board to ensure ethical research practices, and investigators must agree to the terms and conditions of the data use agreement, which includes not distributing the data without permission. All other data are available from the corresponding author upon reasonable request.
