# Supplementary material for: Disease staging of Alzheimer’s disease using a CSF-based biomarker model
Source: Nat Aging. 2024 Mar 21;4(5):694–708. doi: 10.1038/s43587-024-00599-y (PMC11108782; doi:10.1038/s43587-024-00599-y)
Supplement: Supplementary file 1 — Supplementary Methods: Sensitivity analyses on model creation. Supplementary Data: Supplementary Table 1: Demographic characteristics by diagnosis. Supplementary Table 2: Vascular information from BioFINDER-2 participants. Supplementary Table 3: Diagnosis description of non-AD patients in the BioFINDER-2 cohort. Supplementary Table 4: Characteristics of BioFINDER-2 participants with follow-up CSF data. Supplementary Table 5: Statistics of each CSF biomarker and their differences by CSF stage. Supplementary Fig. 1: Description of CSF staging model by vascular risk factors. Supplementary Fig. 2: Description of CSF staging model by vascular pathologies observed in MRI. Supplementary Table 6: Comparison of BioFINDER-2 participants with and without follow-up CSF data. Supplementary Table 7: Statistics of AD biomarkers and their differences by CSF stage. Supplementary Table 8: Statistics of tau-PET binding in different regions and their differences by CSF stage. Supplementary Table 9: Statistics of cognitive composites and their differences by CSF stage. Supplementary Table 10: CSF stages for predicting predicting A/T status and as a diagnostic tool. Supplementary Fig. 3: CSF stages for predicting PET stages. Supplementary Fig. 4: CSF stages for predicting clinical stages. Supplementary Table 11: Characteristics of BioFINDER-2 participants with follow-up AD biomarkers. Supplementary Table 12: Statistics of AD biomarkers longitudinal rates of change and their differences by CSF stage. Supplementary Table 13: CSF stages for predicting disease progression. Supplementary Table 14: Characteristics of Knight ADRC participants with follow-up CSF data. Supplementary Fig. 5: Cross-correlation among biomarkers. Supplementary Fig. 6: Confusion matrix of the ordering of the model when all np-tau fragments are included. Supplementary Fig. 7: Creation of the model in 10 random samples. Supplementary Fig. 8: Simulation of two-subtype models with decreasing prevalence. Supplementary Fig. [file 43587_2024_599_MOESM1_ESM.pdf]

nature aging

Article https://doi.org/10.1038/s43587-024-00599-y

Disease staging of Alzheimer's disease using a CSF-based biomarker model

In the format provided by the authors and unedited

<u>**Supplementary Methods:**</u>

Sensitivity analyses on model creation

<u>**Supplementary Data:**</u>

Supplementary Table 1: Demographic characteristics by diagnosis
Supplementary Table 2: Vascular information from BioFINDER-2 participants
Supplementary Table 3: Diagnosis description of non-AD patients in the BioFINDER-2 cohort
Supplementary Table 4: Characteristics of BioFINDER-2 participants with follow-up CSF data
Supplementary Table 5: Statistics of each CSF biomarker and their differences by CSF stage
Supplementary Figure 1: Description of CSF staging model by vascular risk factors
Supplementary Figure 2: Description of CSF staging model by vascular pathologies observed in MRI
Supplementary Table 6: Comparison of BioFINDER-2 participants with and without follow-up CSF data
Supplementary Table 7: Statistics of AD-biomarkers and their differences by CSF stage
Supplementary Table 8: Statistics of tau-PET binding in different regions and their differences by CSF stage
Supplementary Table 9: Statistics of cognitive composites and their differences by CSF stage
Supplementary Table 10: CSF stages for predicting predicting A/T status and as a diagnostic tool
Supplementary Figure 3: CSF stages for predicting PET stages
Supplementary Figure 4: CSF stages for predicting clinical stages
Supplementary Table 11: Characteristics of BioFINDER-2 participants with follow-up AD biomarkers
Supplementary Table 12: Statistics of AD-biomarkers longitudinal rates of change and their differences by CSF stage
Supplementary Table 13: CSF stages for predicting disease progression
Supplementary Table 14: Characteristics of Knight-ADRC participants with follow-up CSF data
Supplementary Figure 5: Cross-correlation among biomarkers

Supplementary Figure 6: Confusion matrix of the ordering of the model when all np-tau fragments are included

Supplementary Figure 7: Creation of the model in ten random samples

Supplementary Figure 8: Simulation of two subtypes models with decreasing prevalence

Supplementary Figure 9: Simulation of three subtypes models with decreasing prevalence

Supplementary Figure 10: Creation and optimization of the model in the Knight ADRC cohort

Supplementary Methods:

Sensitivity analyses on model creation

We had available data of 5 non-phosphorylated peptides in total (151-155, 181-190, 195-210, 212-221 and 226-230). All these biomarkers were very highly correlated with one another (Pearson's r≥0.98, see Supplementary Figure 5). We hypothesized that including the different np-tau variants would give us a model without additional information. Nonetheless, as a sensitivity analyses, we derived a model including the biomarkers from the final model together will all np-tau variants. This resulted in a model in which all the original biomarkers had the same ordering, with a very high probability, but with a very low probability in the ordering of the different np-tau variants indicating that they change at approximately the same time (see Supplementary Figure 6). Therefore, we decided to include only one in the original model to reduce the number of markers included, acknowledging that they did not add significant information. The selection of this particular fragment was based on the decision to avoid including np-tau peptides that were already included in the model through the ratios of phosphorylated/np-tau measures. Given that the original (prior optimization) model included pTau181/T181 (using np-tau at 181-190), pTau205/T205 (using np-tau at 195-210), pTau217/T217 (using np-tau at 212-221) and pT231/T231 (using np-tau at 226-230), we decided to use the only peptide that was not already in the model (np-tau at 151-155). Nonetheless, as the final model did not include the np-tau at 181-190 nor at 226-230, we redid our model using them instead of the original 151-155, finding nearly identical results (across all models, less than 2% discordance).

To compare our final model to a model created by chance, we also we also derived the model in ten different random samples (random shuffle per biomarker without replacement) including the same number of participants and biomarkers than our final model. In all these random samples, we found that the best model was that having two subtypes, both based in lower CVIC and higher log-likelihood (Supplementary Figure 7A-B). Furthermore, we found that the models created from random measures performed significantly worse on explaining the random data than the original model created based on real biological relationships. This was observed by higher CVIC and lower log-likelihood in the models created from random data compared to our original model (Supplementary Figure 7C). This result strongly supports that our model is significantly better compared to what could be obtained by chance.

Finally, we aimed to assess the minimal size of the subtypes needed to be detected with the sample size available. To this aim, we simulated several datasets with two and three underlying subtypes, respectively, with decreasing proportion of participants in the

smaller subtypes. For this, we used the function *generate_random_mixture_sustain_model* (https://github.com/ucl-pond/pySuStaIn/blob/master/sim/simfuncs.py), similarly as in [1]. In brief, this function randomly creates a dataset with the selected number of participants (n=290 in our case to select only Aβ+ participants), number of biomarkers (5), and number of subtypes. For the two subtypes models we used the following set of proportions: 60%-40%, 70-30%, 80-20%, 90-10%, 95%-5% and 98-2%. For the three subtypes we kept a main subtype and decreased the proportion of the other two, resulting in the following proportions: 40%-30%-30%, 60-20%-20%, 70%-15%-15%, 80%-10%-10%, 90%-5%-5% and 95%-2.5%-2.5%. We then run SuStain on this data as in our original dataset and derived the optimal number of subtypes based on CVIC and log-likelihood. When simulating two subtypes, SuStaIn was able to discern these two subtypes until the smallest subtype included a 5% of the sample, but not with smaller numbers (see Supplementary Figure 8). For the three subtypes models, SuStaIn selected three subtypes also until the smaller subtypes included 5% of the sample (see Supplementary Figure 9). Based on these results, the lack of different subtypes in our main analysis seems to be due to a lack of any other subtype, unless other trajectories exist but are extremely low prevalent.

Supplementary Data:

	BioFINDER-2 cohort						Knight ADRC cohort					
	**All (n=426)**	**CU- (n=80)**	**CU+ (n=79)**	**MCI+ (n=88)**	**ADD+ (n=100)**	**nonAD (n=79)**	**All (n=222)**	**CU- (n=84)**	**CU+ (n=98)**	**Very mild AD (n=24)**	**AD dementia (n=9)**	**Other dementias (n=7)**
**Age, years**	71.5 (8.5)	70.7 (9.5)	71.1 (9.5)	72.0 (7.4)	73.0 (6.9)	70.2 (9.0)	71.2 (7.7)	67.6 (7.2)	73.2 (7.4)	73.4 (6.4)	74.1 (7.6)	75.0 (7.2)
**Women, n(%)**	211 (49.5%)	39 (48.8%)	40 (50.6%)	38 (43.2%)	56 (56.0%)	38 (48.1%)	112 (50.5%)	39 (46.4%)	56 (57.1%)	9 (37.5%)	4 (44.4%)	4 (57.1%)
***APOE-ε4* carriership, n(%)[a]**	246 (57.7%)	26 (32.5%)	58 (73.4%)	62 (70.5%)	74 (74.0%)	26 (32.9%)	99 (44.6%)	18 (21.4%)	56 (57.1%)	15 (62.5%)	6 (66.7%)	4 (57.1%)
**Years of education[b]**	12.3 (3.8)	12.0 (3.2)	12.2 (3.4)	12.7 (4.5)	12.0 (4.0)	12.7 (3.5)	16.3 (2.5)	16.5 (2.3)	16.5 (2.4)	14.9 (2.8)	14.7 (2.6)	17.7 (2.2)
**Amyloid-PET, Centiloids[c]**	37.3 (44.2)	-4.48 (9.50)	41.8 (36.0)	69.6 (36.0)	115 (23.3)	5.67 (23.3)	44.0 (41.2)	7.6 (11.2)	57.7 (33.3)	83.6 (23.7)	119 (38.3)	57.9 (46.8)
**Tau-PET, SUVR[d]**	1.53 (0.61)	1.16 (0.08)	1.23 (0.21)	1.50 (0.45)	2.38 (0.58)	1.16 (0.10)	1.24 (0.22)	1.13 (0.08)	1.22 (0.13)	1.51 (0.38)	1.72 (0.25)	1.23 (0.12)
**Cortical thickness, mm[e]**	2.46 (0.16)	2.56 (0.09)	2.55 (0.12)	2.46 (0.13)	2.32 (0.15)	2.46 (0.18)	2.52 (0.16)	2.59 (0.12)	2.53 (0.14)	2.39 (0.17)	2.29 (0.15)	2.35 (0.19)
**CSF NfL[f]**	245 (175)	147 (79.1)	189 (153)	223 (128)	316 (180)	333 (222)	1000 (578)	740 (313)	1010 (489)	1660 (974)	1360 (404)	1230 (569)
**Cognitive composite[#g]**	-1.62 (2.03)	0.06 (0.76)	-0.26 (0.78)	-1.88 (0.72)	-4.34 (1.72)	-1.69 (2.03)	0.44 (1.11)	-0.01 (0.72)	0.36 (0.75)	2.01 (0.96)	3.86 (2.36)	1.12 (1.40)
**Progressed to MCI[†h]**	11 (2.6%)	0 (0%)	11 (13.9%)	-	-	-	41 (18.5%)	8 (9.5%)	33 (33.7%)	-	-	-
**Progressed to ADD+[¥i]**	41 (9.6%)	0 (0%)	3 (3.8%)	38 (43.2%)	-	-	30 (14.5%)	0 (0%)	14 (14.3%)	16 (66.7%)	-	-

Supplementary Table 1: Participants' characteristics

Data is shown as mean(SD) unless otherwise stated. Participants are divided by clinical diagnosis and amyloid status based on their CSF Aβ42/40 levels (Aβ+: <0.080 for BioFINDER-2 and Aβ+: <0.0673 for Knight ADRC). In BioFINDER-2, only participants who progressed to MCI or dementia patients due to AD etiology were considered to progress. In Knight ADRC, Very mild AD dementia patients had a CDR=0.5 and mild AD dementia patients had a CDR≥1, both with AD as etiology. Other dementias group includes participants with CDR>0 with non-AD etiology. Only participants who progressed to CDR≥0.5 or CDR≥1 due to AD etiology were considered to progress. [#]Cognitive composite was mPACC for BioFINDER-2 and a global cognitive composite in Knight ADRC. [†]For Knight ADRC represents progression to CDR≥0.5. [¥] For Knight ADRC represents progression to CDR≥1.

[a], 1 participant missing in both cohorts; [b], 4 participants missing in BioFINDER-2; [c], 175 participants missing in BioFINDER-2; [d], 9 and 3 participants missing in BioFINDER-2 and Knight ADRC, respectively; [e], 6 participants missing in BioFINDER-2; [f], 4 and 5 participants missing in BioFINDER-2 and Knight ADRC, respectively; [g], 36 and 2 participants missing in BioFINDER-2 and Knight ADRC, respectively; [h], 4 participants missing in Knight ADRC; [i], 8 participants missing in Knight ADRC.

Abbreviations: Aβ, amyloid-β; AD, Alzheimer's disease; ADD+, Alzheimer's disease dementia amyloid positive; CDR, clinical dementia rating; CU-, cognitively unimpaired amyloid negative; CU+, cognitively unimpaired amyloid positive; CSF, cerebrospinal fluid; MCI, mild cognitive impairment amyloid positive; mPACC, modified preclinical Alzheimer's cognitive composite; nonAD, non-Alzheimer's related disease; PET, positron emission tomography; SD, standard deviation; SUVR, standardized uptake value ratio.

	All (n=426)	CU- (n=80)	CU+ (n=79)	MCI+ (n=88)	ADD+ (n=100)	nonAD (n=79)
**Hypertension**	137 (32.2%)	26 (32.5%)	24 (30.4%)	26 (29.5%)	32 (32.0%)	29 (36.7%)
**Hyperlipidemia**	42 (9.9%)	13 (16.3%)	12 (15.2%)	2 (2.3%)	8 (8.0%)	7 (8.9%)
**Diabetes**	42 (9.9%)	9 (11.3%)	6 (7.6%)	8 (9.1%)	10 (10.0%)	9 (11.4%)
**WML Fazekas**						
0	30 (7.0%)	6 (7.5%)	8 (10.1%)	2 (2.3%)	4 (4.0%)	10 (12.7%)
1	224 (52.6%)	48 (60.0%)	38 (48.1%)	45 (51.1%)	47 (47.0%)	46 (58.2%)
2	122 (28.6%)	16 (20.0%)	21 (26.6%)	31 (35.2%)	35 (35.0%)	19 (24.1%)
3	40 (9.4%)	7 (8.8%)	7 (8.9%)	10 (11.4%)	14 (14.0%)	2 (2.5%)
**Lacunes**	28 (6.6%)	4 (5.0%)	4 (5.1%)	9 (10.2%)	4 (4.0%)	7 (8.9%)
**Ischemic infarcts**	29 (6.8%)	7 (8.8%)	3 (3.8%)	4 (4.5%)	11 (11.0%)	4 (5.1%)
**Hemorrhages**	4 (0.9%)	0 (0%)	0 (0%)	0 (0%)	4 (4.0%)	0 (0%)
**Microbleeds**	73 (17.1%)	9 (11.3%)	11 (13.9%)	21 (23.9%)	28 (28.0%)	4 (5.1%)
**Siderosis**	7 (1.6%)	0 (0%)	1 (1.3%)	3 (3.4%)	3 (3.0%)	0 (0%)
**Vascular weight**						
0	240 (56.3%)	52 (65.0%)	49 (62.0%)	39 (44.3%)	44 (44.0%)	56 (70.9%)
1	123 (28.9%)	19 (23.8%)	21 (26.6%)	35 (39.8%)	31 (31.0%)	17 (21.5%)
2	45 (10.6%)	9 (11.3%)	8 (10.1%)	8 (9.1%)	17 (17.0%)	3 (3.8%)
3	15 (3.5%)	0 (0%)	0 (0%)	5 (5.7%)	7 (7.0%)	3 (3.8%)
4	3 (0.7%)	0 (0%)	1 (1.3%)	1 (1.1%)	1 (1.0%)	0 (0%)

Supplementary Table 2: Vascular information from BioFINDER-2 participants

Data shows presence of pathology [n (%)]. Microbleeds are assessed as positive if there were more than 1 microbleed regardless of location. WML were considered pathological with a Fazekas score ≥2. Vascular weight was calculated as the sum of co-occurrent vascular pathologies (WML, lacunes, ischemic infarcts, hemorrhages, microbleeds and siderosis). High vascular pathology burden was considered as those with presence of three or more pathological measures.

Abbreviations: ADD+, Alzheimer's disease dementia amyloid positive; CU-, cognitively unimpaired amyloid negative; CU+, cognitively unimpaired amyloid positive; MCI+, mild cognitive impairment amyloid positive; nonAD, non-Alzheimer's related disease; WML, white matter lesions.

	nonAD Aβ- (n=56)	nonAD Aβ+ (n=23)
**CBS**	2 (3.6%)	2 (8.7%)
**FTD**	13 (23.2%)	4 (17.4%)
**MSA**	1 (1.8%)	0 (0%)
**Parkinsonism/PD**	19 (33.9%)	8 (34.8%)
**PPA**	4 (7.1%)	4 (17.4%)
**PSP**	15 (26.8%)	5 (21.7%)

Supplementary Table 3: Diagnosis description of non-AD patients in the BioFINDER-2 cohort

Abbreviations: Aβ, amyloid-β; CBS, corticobasal syndrome; FTD, frontotemporal disease; MSA, multiple system atrophy; PD, Parkinson's disease; PPA, primary progressive aphasia; PSP, progressive supranuclear palsy.

CSF stage at baseline	All (n=220)	CU- (n=80)	CU+ (n=49)	MCI+ (n=46)	ADD+ (n=28)	nonAD (n=17)
**Age, years**	71.4 (8.80)	70.7 (9.46)	72.8 (7.71)	71.2 (9.06)	74.1 (7.67)	67.5 (8.45)
**Women, n(%)**	107 (48.6%)	39 (48.8%)	23 (46.9%)	24 (52.2%)	15 (53.6%)	6 (35.3%)
***APOE-ε4* carriershp, n(%)**	121 (55.0%)	26 (32.5%)	36 (73.5%)	33 (71.7%)	20 (71.4%)	6 (35.3%)
**Years of education**	12.2 (3.9)	12.0 (3.2)	12.0 (3.5)	12.9 (5.1)	11.5 (3.9)	12.6 (4.5)
**Years between baseline and follow-up**	2.05 (0.22)	1.91 (0.11)	2.04 (0.23)	2.16 (0.24)	2.19 (0.17)	2.17 (0.19)
**Stage at baseline, n(%)**						
**0**	87 (39.5%)	74 (92.5%)	0 (0%)	0 (0%)	0 (0%)	13 (76.5%)
**1**	15 (6.8%)	2 (2.5%)	10 (20.4%)	3 (6.5%)	0 (0%)	0 (0%)
**2**	28 (12.7%)	3 (3.8%)	15 (30.6%)	9 (19.6%)	0 (0%)	1 (5.9%)
**3**	27 (12.3%)	1 (1.3%)	12 (24.5%)	10 (21.7%)	2 (7.1%)	2 (11.8%)
**4**	35 (15.9%)	0 (0%)	10 (20.4%)	15 (32.6%)	9 (32.1%)	1 (5.9%)
**5**	28 (12.7%)	0 (0%)	2 (4.1%)	9 (19.6%)	17 (60.7%)	0 (0%)
**Stage at follow-up, n(%)**						
**0**	89 (40.5%)	76 (95.0%)	0 (0%)	0 (0%)	0 (0%)	13 (76.5%)
**1**	10 (4.5%)	2 (2.5%)	5 (10.2%)	3 (6.5%)	0 (0%)	0 (0%)
**2**	25 (11.4%)	1 (1.3%)	20 (40.8%)	3 (6.5%)	0 (0%)	1 (5.9%)
**3**	23 (10.5%)	1 (1.3%)	9 (18.4%)	11 (23.9%)	0 (0%)	2 (11.8%)
**4**	41 (18.6%)	0 (0%)	14 (28.6%)	15 (32.6%)	11 (39.3%)	1 (5.9%)
**5**	32 (14.5%)	0 (0%)	1 (2.0%)	14 (30.4%)	17 (60.7%)	0 (0%)

Supplementary Table 4: Characteristics of BioFINDER-2 participants with follow-up CSF data

As CSF Aβ42/40 levels were not available for any participant we imputed this data with their baseline levels. Data is shown as mean (SD) unless otherwise stated.

Abbreviations: Aβ, amyloid-β; AD, Alzheimer's disease; ADD+, Alzheimer's disease dementia amyloid positive; CU-, cognitively unimpaired amyloid negative; CU+, cognitively unimpaired amyloid positive; CSF, cerebrospinal fluid; MCI+, mild cognitive impairment amyloid positive; nonAD, non-Alzheimer's related disease; SD, standard deviation.

Biomarker	Mean (SD) CSF stage 0	Mean (SD) CSF stage 1	Mean (SD) CSF stage 2	Mean (SD) CSF stage 3	Mean (SD) CSF stage 4	Mean (SD) CSF stage 5
**BioFINDER-2**						
Aβ42/40	0.02 (0.83)	2.84 (0.7)	3.59 (1.06)	3.51 (1.42)	4.45 (0.94)	4.91 (0.77)
pT217/T217	-0.3 (0.64)	0.25 (0.64)	3.39 (1.38)	5.47 (2.69)	10.04 (3.39)	13.77 (4.09)
pT205/T205	-0.15 (1.13)	-0.28 (0.95)	0.24 (0.99)	3.64 (1.52)	5.49 (2.45)	5.8 (3.26)
MTBR-tau243	-0.24 (0.95)	-0.07 (0.74)	0.31 (1.02)	0.57 (0.92)	5.71 (2.58)	13.33 (7.37)
np-tau	-0.17 (1.05)	0.38 (1.2)	0.56 (1.19)	0.23 (0.89)	1.54 (0.72)	5.08 (2.11)
**Knight-ADRC**						
Aβ42/40	-0.23 (0.94)	3.26 (1.42)	4.10 (1.15)	4.07 (1.34)	4.57 (1.41)	5.39 (0.81)
pT217/T217	-0.29 (0.38)	0.08 (0.30)	3.70 (2.65)	5.81 (3.05)	9.76 (2.66)	10.05 (3.19)
pT205/T205	-0.16 (0.78)	-0.61 (1.14)	0.20 (0.63)	2.72 (1.25)	5.59 (2.06)	4.27 (2.59)
MTBR-tau243	-0.17 (0.74)	0.47 (1.06)	0.80 (0.69)	0.85 (0.85)	5.36 (2.48)	6.38 (3.61)
np-tau	-0.13 (0.92)	0.78 (1.19)	1.06 (1.16)	0.83 (0.97)	0.85 (0.35)	3.33 (1.48)

Supplementary Table 5: Statistics of each CSF biomarker and their differences by CSF stage

Mean (SD) z-scores by CSF stages are shown calculated from a group of cognitively unimpaired amyloid negative participants, independently in each cohort. Aβ42/40 z-scores were inverted to obtain higher values for higher abnormality. Letters represent statistically significant difference with: a, CSF stage 0; b, CSF stage 1; c, CSF stage 2; d, CSF stage 3; e, CSF stage 4; f, CSF stage 5.

Abbreviations: Aβ, amyloid-β; CSF, cerebrospinal fluid; MTBR, microtubule binding region; np-tau, non-phosphorylated mid-region tau; pT, phosphorylated tau; SD, standard deviation.

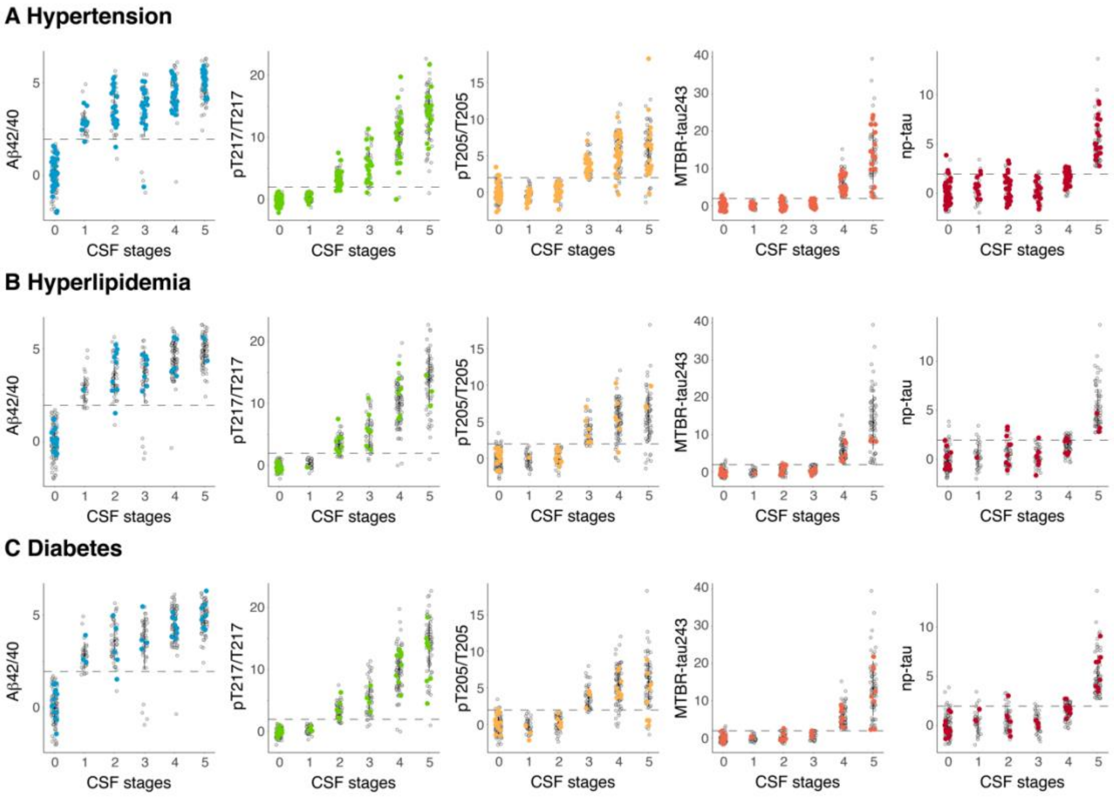

Supplementary Figure 1: Description of CSF staging model by vascular risk factors

Levels of CSF biomarkers by CSF stages are shown for participants with hypertension (A), hyperlipidemia (B) and diabetes. We can observe that those participants with a particular vascular risk factor (colored) follow the same pattern of biomarker levels than those without it (in grey). CSF levels are z-scored based on a group of CU- participants (n=63) and all increases represent increase in abnormality. Horizontal line is drawn at z-score=1.96 which represents 95%CI of the reference group (CU-). CSF stage 0 represent being classified as normal by the model. Black dots and vertical lines represent mean and SD by CSF stage, respectively.

Abbreviations: Aβ, amyloid-β; CI, confidence interval; CU-, cognitively unimpaired amyloid negative; CSF, cerebrospinal fluid; MTBR, microtubule binding region; np-tau, non-phosphorylated mid-region tau; pT, phosphorylated tau.

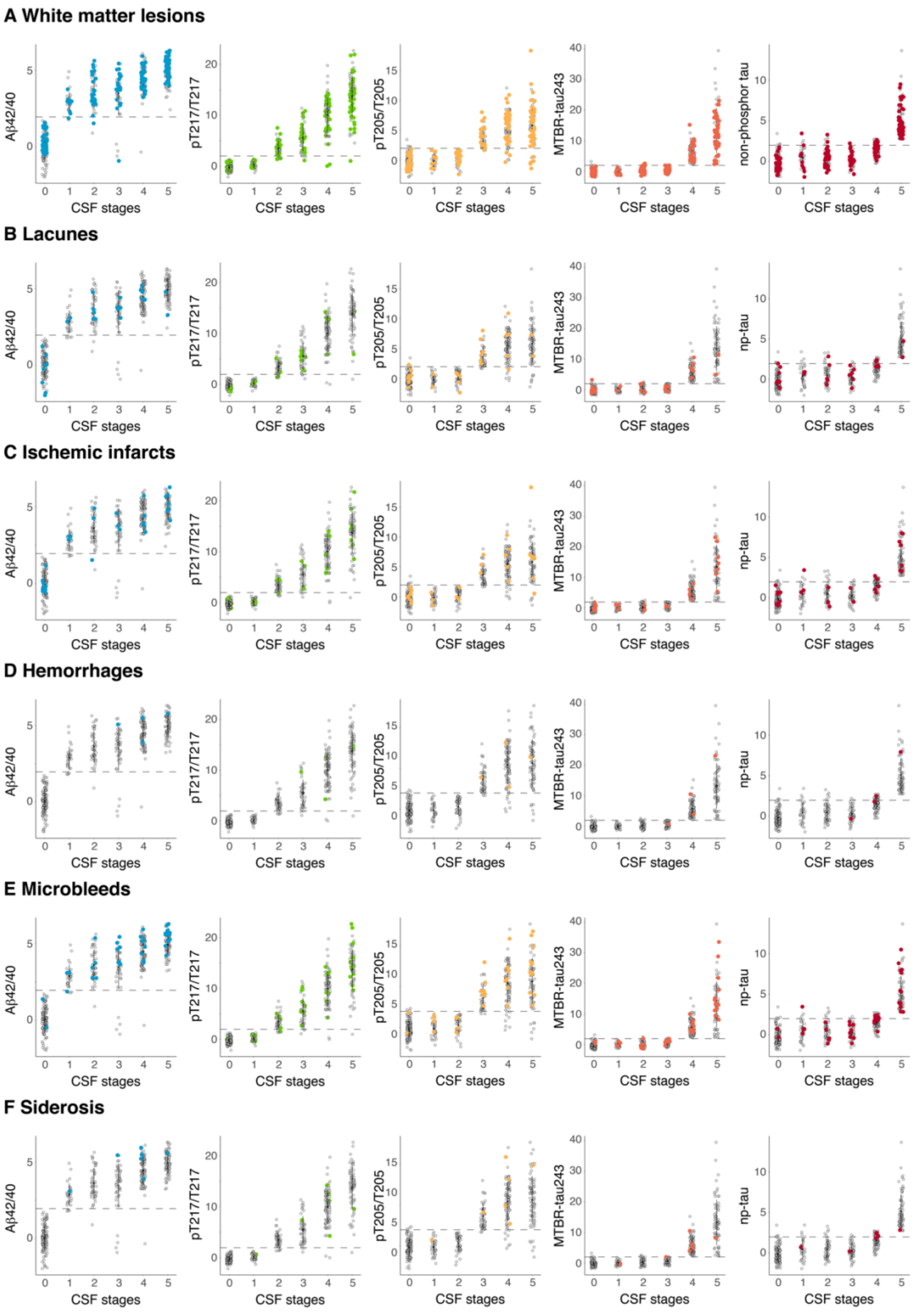

Supplementary Figure 2: Description of CSF staging model by vascular pathologies observed in MRI

Levels of CSF biomarkers by CSF stages are shown for participants vascular pathologies measured in the MRI by a radiologist. We can observe that those participants with a particular vascular risk factor (colored) follow the same pattern of biomarker levels than

those without it (in grey). CSF levels are z-scored based on a group of CU- participants (n=63) and all increases represent increase in abnormality. Horizontal line is drawn at z-score=1.96 which represents 95%CI of the reference group (CU-). CSF stage 0 represent being classified as normal by the model. Black dots and vertical lines represent mean and SD by CSF stage, respectively. Microbleeds are assessed as positive if there were more than 1 microbleed regardless of location. White matter lesions were considered pathological with a Fazekas score ≥2.

Abbreviations: Aβ, amyloid-β; CI, confidence interval; CU-, cognitively unimpaired amyloid negative; CSF, cerebrospinal fluid; MTBR, microtubule binding region; np-tau, non-phosphorylated mid-region tau; pT, phosphorylated tau.

	Baseline only	Longitudinal information	p
**Age, years**	71.5 (8.10)	71.4 (8.80)	0.965
**Women, n(%)**	104 (50.5%)	107 (48.6%)	0.776
APOE-ε4 **carriershp, n(%)**[a]	125 (60.7%)	121 (55.0%)	0.251
**Years of education**[b]	12.4 (3.59)	12.2 (3.92)	0.471
**Amyloid-PET, Centiloids**[c]	63.4 (43.5)	24.9 (38.9)	<0.001
**Tau-PET, SUVR**[d]	1.68 (0.688)	1.40 (0.492)	<0.001
**Cortical thickness, mm**[e]	2.42 (0.178)	2.50 (0.138)	<0.001
**CSF NfL**[f]	282 (170)	210 (172)	<0.001
**mPACC**[g]	-2.28 (2.07)	-1.06 (1.82)	<0.001
**Progressed to MCI**	2 (1.0%)	9 (4.1%)	0.085
**Progressed to ADD+**	17 (8.3%)	24 (10.9%)	0.444

Supplementary Table 6: Comparison of BioFINDER-2 participants with and without follow-up CSF data

Data is shown as mean(SD) unless otherwise stated. Participants are divided by those with and without follow-up CSF data. Only participants who progressed to MCI or dementia patients due to AD etiology were considered to progress.

[a], 1 participant missing; [b], 4 participants missing; [c], 175 participants missing; [d], 9 participants missing; [e], 6 participants missing; [f], 4 participants missing; [g], 36 participants missing.

Abbreviations: Aβ, amyloid-β; AD, Alzheimer's disease; ADD+, Alzheimer's disease dementia amyloid positive; CU-, cognitively unimpaired amyloid negative; CU+, cognitively unimpaired amyloid positive; CSF, cerebrospinal fluid; MCI, mild cognitive impairment amyloid positive; nonAD, non-Alzheimer's related disease; PET, positron emission tomography; ROI, region of interest; SD, standard deviation; SUVR, standardized.

Biomarker	Mean (SD) CSF stage 0	Mean (SD) CSF stage 1	Mean (SD) CSF stage 2	Mean (SD) CSF stage 3	Mean (SD) CSF stage 4	Mean (SD) CSF stage 5
**BioFINDER-2**						
Amyloid-PET	-0.19 (0.58)[b,c,d,e,f]	0.93 (2.12)[a,c,d,e,f]	4.01 (2.71)[a,b,d,e,f]	5.94 (2.64)[a,b,c,e,f]	8.3 (3.03)[a,b,c,d]	9.06 (3.17)[a,b,c,d]
Tau-PET	-0.27 (0.92)[d,e,f]	-0.4 (1.12)[d,e,f]	-0.07 (0.9)[d,e,f]	2.02 (3.16)[a,b,c,e,f]	9.25 (7.03)[a,b,c,d,f]	12.67 (8.43)[a,b,c,d,e]
Cortical thickness	-0.22 (0.99)[d,e,f]	0.1 (1.19)[d,e,f]	0.08 (1.23)[d,e,f]	0.88 (1.54)[a,b,c,f]	1.59 (1.69)[a,b,c]	2.07 (1.91)[a,b,c,d]
CSF NfL	-0.15 (0.97)[c,d,e,f]	0.76 (2.81)[e,f]	0.32 (1.64)[a,e,f]	0.70 (1.97)[a,e,f]	1.06 (2.17)[a,b,c,d,f]	1.72 (1.28)[a,b,c,d,e]
mPACC	-0.11 (0.73)[c,d,e,f]	0.17 (0.73)[d,e,f]	0.61 (1.08)[a,d,e,f]	1.53 (1.37)[a,b,c,e,f]	2.6 (1.84)[a,b,c,d,f]	3.65 (1.92)[a,b,c,d,e]
**Knight-ADRC**						
Amyloid-PET	-0.15 (0.97)[c,d,e,f]	0.84 (1.38)[d,e,f]	2.88 (2.47)[a,d,e,f]	4.89 (2.95)[a,b,c,e,f]	7.66 (3.81)[a,b,c,d]	6.52 (3.13)[a,b,c,d]
Tau-PET	-0.14 (1.1)[d,e,f]	-0.32 (0.84)[e,f]	0.3 (1.2)[e,f]	0.56 (1.1)[a,e,f]	5.89 (3.62)[a,b,c,d]	4.54 (4.75)[a,b,c,d]
Cortical thickness	-0.18 (1.02)[d,e,f]	-0.6 (0.22)[e,f]	-0.16 (0.75)[d,e,f]	0.55 (1.16)[a,c,e,f]	2.42 (0.90)[a,b,c,d,f]	1.38 (1.58)[a,b,c,d,e]
CSF NfL	-0.22 (0.92)[c,d,e,f]	0.35 (0.83)	0.51 (1.41)[a,d,f]	1.19 (1.92)[a,c,f]	0.81 (0.90)[a]	2.08 (2.65)[a,c,d]
Global cognitive composite	-0.05 (0.71)[c,d,f]	-0.42 (0.49)[f]	0.39 (0.74)[a]	0.69 (1.01)[a]	0.65 (0.96)	1.25 (1.66)[a,b]

Supplementary Table 7: Statistics of AD-biomarkers and their differences by CSF stage

Mean (SD) z-scores by CSF stages are shown calculated from a group of cognitively unimpaired amyloid negative participants, independently in each cohort. Differences among CSF stages were calculated using pairwise Wilcoxon test. P-values were FDR-corrected for multiple comparisons. Cortical thickness and mPACC z-scores were inverted to obtain higher values for higher abnormality. Letters represent statistically significant difference with: a, CSF stage 0; b, CSF stage 1; c, CSF stage 2; d, CSF stage 3; e, CSF stage 4; f, CSF stage 5.

Abbreviations: Aβ, amyloid-β; CSF, cerebrospinal fluid; FDR, false-discovery rate; mPACC, modified preclinical Alzheimer's cognitive composite; MTBR, microtubule binding region; NfL, neurofilament light; PET, positron emission tomography; pT, phosphorylated tau; SD, standard deviation.

ROI	Mean (SD) CSF stage 0	Mean (SD) CSF stage 1	Mean (SD) CSF stage 2	Mean (SD) CSF stage 3	Mean (SD) CSF stage 4	Mean (SD) CSF stage 5
**BioFINDER-2**						
Braak I-II	-0.14 (1.00)[c,d,e,f]	-0.23 (1.15)[d,e,f]	0.25 (1.18)[a,d,e,f]	2.26 (2.14)[a,b,c,e,f]	6.58 (3.31)[a,b,c,d,f]	7.49 (3.77)[a,b,c,d,e]
Braak III-IV	-0.28 (0.92)[d,e,f]	-0.41 (1.12)[d,e,f]	-0.10 (0.9)[d,e,f]	1.97 (3.24)[a,b,c,e,f]	9.37 (7.35)[a,b,c,d,f]	12.92 (8.76)[a,b,c,d,e]
Braak V-VI	-0.19 (0.92)[d,e,f]	-0.55 (1.13)[d,e,f]	-0.37 (0.76)[d,e,f]	0.77 (2.09)[a,b,c,e,f]	4.58 (4.98)[a,b,c,d]	5.93 (5.32)[a,b,c,d]
**Knight-ADRC**						
Braak I-II	-0.07 (1.03)[c,d,e,f]	-0.54 (0.46)[d,e,f]	0.47 (1.47)[a,e,f]	1 (1.74)[a,b,e,f]	3.48 (1.61)[a,b,c,d]	3.70 (2.35)[a,b,c,d]
Braak III-IV	-0.14 (1.10)[d,e,f]	-0.30 (0.85)[e,f]	0.28 (1.17)[e,f]	0.53 (1.08)[a,e,f]	5.94 (3.78)[a,b,c,d]	4.51 (4.85)[a,b,c,d]
Braak V-VI	-0.08 (1.07)[e,f]	-0.30 (0.47)[f]	0.26 (0.99)[f]	0.24 (0.85)[f]	1.92 (2.9)[a]	2.01 (2.91)[a,b,c,d]

Supplementary Table 8: Statistics of tau-PET binding in different regions and their differences by CSF stage

Mean(SD) z-scores by CSF stages are shown calculated from a group of cognitively unimpaired amyloid negative participants, independently in each cohort. Differences among CSF stages were calculated using pairwise Wilcoxon test. P-values were FDR-corrected for multiple comparisons. Letters represent statistically significant difference with: a, CSF stage 0; b, CSF stage 1; c, CSF stage 2; d, CSF stage 3; e, CSF stage 4; f, CSF stage 5.

Abbreviations: CSF, cerebrospinal fluid; FDR, false-discovery rate; PET, positron emission tomography; ROI, region of interest; SD, standard deviation.

Biomarker	Mean (SD) CSF stage 0	Mean (SD) CSF stage1	Mean (SD) CSF stage 2	Mean (SD) CSF stage 3	Mean (SD) CSF stage 4	Mean (SD) CSF stage 5
**BioFINDER-2**						
mPACC	-0.11 (0.73)[c,d,e,f]	0.17 (0.73)[d,e,f]	0.61 (1.08)[a,d,e,f]	1.53 (1.37)[a,b,c,e,f]	2.6 (1.84)[a,b,c,d,f]	3.65 (1.92)[a,b,c,d,e]
Memory	-0.22 (0.89)[b,c,d,e,f]	0.27 (1.07)[a,d,e,f]	0.58 (1.25)[a,d,e,f]	1.17 (1.31)[a,b,c,e,f]	2.18 (1.24)[a,b,c,d,f]	2.7 (0.98)[a,b,c,d,e]
Executive function	-0.29 (0.78)[c,d,e,f]	-0.24 (1.13)[c,d,e,f]	0.49 (1.27)[a,b,e,f]	1.04 (1.8)[a,b,f]	1.79 (2.16)[a,b,c]	2.15 (1.58)[a,b,c,d]
Language	-0.20 (0.86)[c,d,e,f]	-0.17 (0.71)[d,e,f]	0.28 (1.02)[a,e,f]	0.85 (1.3)[a,b,f]	1.22 (1.18)[a,b,c,f]	1.91 (1.32)[a,b,c,d,e]
Visuospatial	-0.15 (0.69)[d,e,f]	0.00 (1.17)[e,f]	0.06 (0.96)[e,f]	0.54 (1.34)[a]	1.11 (2.63)[a,b,c]	1.59 (2.82)[a,b,c]
MMSE	-0.14 (0.91)[c,d,e,f]	0.19 (1.26)[d,e,f]	0.68 (1.48)[a,d,e,f]	2.17 (2.8)[a,b,c,e,f]	4.2 (3.98)[a,b,c,d,f]	6.15 (4.51)[a,b,c,d,e]
**Knight-ADRC**						
Global cognitive composite	-0.05 (0.71)[c,d,f]	-0.42 (0.49)[f]	0.39 (0.74)[a]	0.69 (1.01)[a]	0.65 (0.96)	1.25 (1.66)[a,b]
Memory	-0.12 (0.98)[c,d,e,f]	-0.13 (0.68)	0.68 (1.04)[a,f]	1.07 (1.53)[a]	1.96 (1.53)[a]	1.84 (1.92)[a,c]
Executive function	-0.01 (1.03)[c,d,e,f]	-0.61 (0.33)[c,d,f]	0.42 (0.95)[a,b]	0.64 (1.18)[a,b]	1.77 (2.58)[a]	1.59 (2.4)[a,b]
Language	-0.05 (0.92)[e,f]	-0.32 (1.43)	0.05 (1.08)[e,f]	0.43 (1.03)[e,f]	1.2 (1.1)[a,c,d]	1.09 (1.19)[a,c,d]
MMSE	-0.02 (1.02)[e,f]	-0.69 (0.00)[e,f]	0.42 (1.27)[e,f]	0.4 (1.8)[e,f]	3.83 (4.37)[a,b,c,d]	3.1 (3.89)[a,b,c,d]

Supplementary Table 9: Statistics of cognitive composites and their differences by CSF stage

Mean(SD) z-scores by CSF stages are shown calculated from a group of cognitively unimpaired amyloid negative participants, independently in each cohort. Differences among CSF stages were calculated using pairwise Wilcoxon test. P-values were FDR-corrected for multiple comparisons. For all tests higher z-scores represent higher abnormality. Letters represent statistically significant difference with: a, CSF stage 0; b, CSF stage 1; c, CSF stage 2; d, CSF stage 3; e, CSF stage 4; f, CSF stage 5.

Abbreviations: Aβ, amyloid-β; CSF, cerebrospinal fluid; FDR, false-discovery rate; MMSE, Mini-Mental state examination; mPACC, modified preclinical Alzheimer's

cognitive composite; MTBR, microtubule binding region; NfL, neurofilament light; PET, positron emission tomography; SD, standard deviation.

	AUC [95%CI] or C-index[95%CI]	CSF stage cut-off (≥)	Sensitivity	Specificity	Accuracy
**BioFINDER-2**					
Amyloid-PET	0.96[0.93,0.98]	2	0.93	0.89	0.92
Tau-PET	0.95[0.93,0.97]	4	0.91	0.92	0.91
A/T PET status	0.95[0.93,0.97]	-	-	-	-
Diagnosis (AD *continuum*)	0.88[0.86,0.91]	-	-	-	-
AD vs non-AD cognitive impairment	0.95[0.93,0.98]	2	0.97	0.75	0.91
**Knight-ADRC**					
Amyloid-PET	0.89[0.85,0.94]	2	0.91	0.81	0.87
Tau-PET	0.94[0.91,0.96]	4	0.92	0.87	0.88
A/T PET status	0.89[0.86,0.92]	-	-	-	-

Supplementary Table 10: CSF stages for predicting predicting A/T status and as a diagnostic tool

ROC curves were used to characterize CSF staging for predicting amyloid-PET (A), tau-PET (T) positivity or as a diagnostic tool (AD vs non-AD cognitive impairment). Maximization of Youden's index was used to find the optimal CSF stage for separating groups. In AD vs non-AD analysis, only impaired participants were included (MCI or dementia). Ordinal logistic regression was used to classify A/T status and diagnosis. A-T+ (n=1 in each cohort) and non-AD participants were excluded from A/T status and diagnosis analyses, respectively. A/T status was categorized based on PET. For Knight-ADRC, CSF stages 0 and 1 were merged for the A/T status analysis due to low number of subjects at CSF stage 1. AUC was used in ROC analyses and C-index was used in ordinal logistic regression as a performance measure. Amyloid-PET was considered positive if SUVR>1.03 (BioFINDER-2) and Centiloid>20 (Knight-ADRC), tau-PET was considered positive if SUVR at meta-temporal ROI (Braak I-IV) was higher than 1.36 (BioFINDER-2) and 1.32 SUVR (Knight-ADRC).

Abbreviations: AD, Alzheimer's disease; A-T+, amyloid-negative tau-positive; AUC, area under the curve; CI, confidence interval; C-index, concordance index; CDR, clinical dementia rating; CSF, cerebrospinal fluid; MCI, mild cognitive impairment; PET, positron

emission tomography; ROC, receiver operating characteristic; ROI, region of interest; SUVR, standardized uptake value ratio.

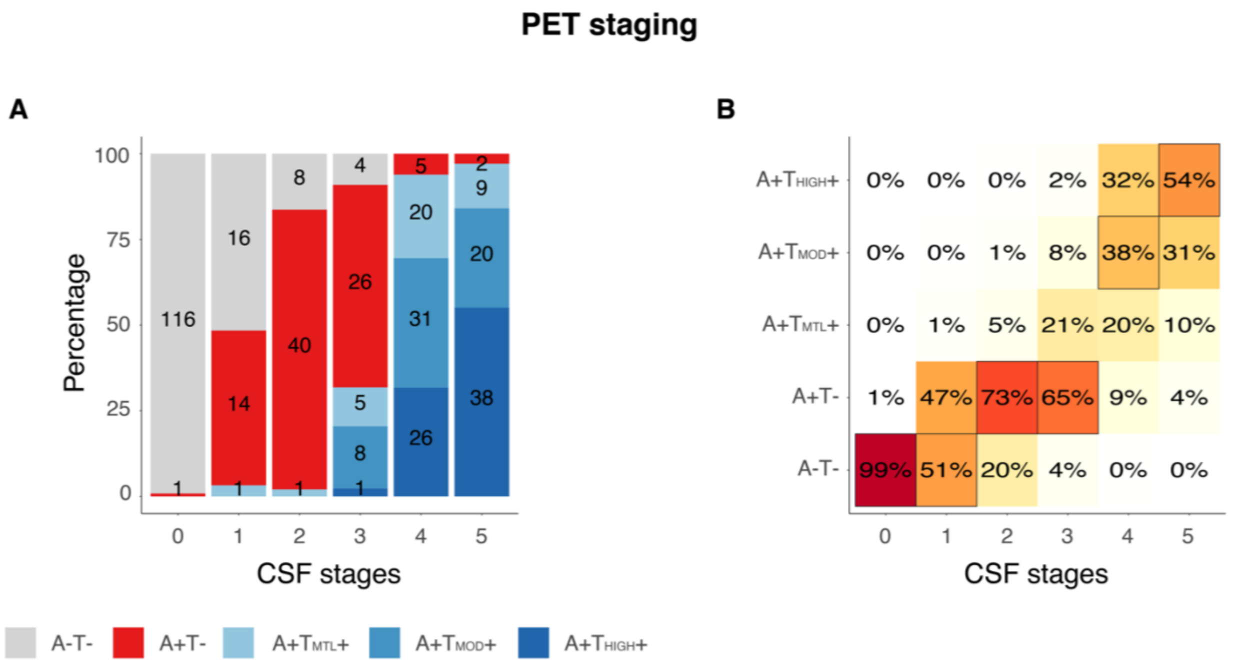

Supplementary Figure 3: CSF stages for predicting PET stages
Barplots represent the number of participants in each category per CSF stage (A). Numbers of participants in each category per CSF stage are shown within the barplots. An ordinal logistic regression was used for predicting PET stages by CSF stages. The heatmap represent the predicted percentage of participants in each outcome category by CSF stage. The most probable (highest percentage) category by CSF stage is framed in black. PET stages included: all negative (A-T-), initial stage (A+T-), early stage (A+T_{MTL}+), intermediate stage (A+T_{MOD}+) and advanced stage (A+T_{HIGH}+). The amyloid status was assessed using amyloid-PET in the neocortex (SUVR>1.03) in all cases except in AD dementia and non-AD cases in which amyloid-PET was not acquired. In these cases, amyloid status was assessed by CSF Aβ42/40 (<0.080). Tau positivity in the MTL was assessed using a ROI including the entorhinal cortex and the amygdala as previously explained (SUVR>1.34)[2]. Finally, tau status in the neocortex was assessed using the MUBADA ROI[3], which is a voxel-wise map that optimally discriminates between Aβ-positive clinically defined MCI and AD dementia patients against Aβ-negative cases (Moderate: 1.10≤SUVR<1.46; High: SUVR≥1.46)[4].

Abbreviations: Aβ, amyloid-β; AD, Alzheimer's disease; ADD+, Alzheimer's disease dementia amyloid-positive; CSF, cerebrospinal fluid; CU, cognitively unimpaired; MCI,

mild cognitive impairment; MTL, medial temporal lobe; MUBADA, multi-block barycentric discriminant analysis; ROI, region of interest; SCD, subjective cognitive decline; SUVR, standardized uptake value ratio.

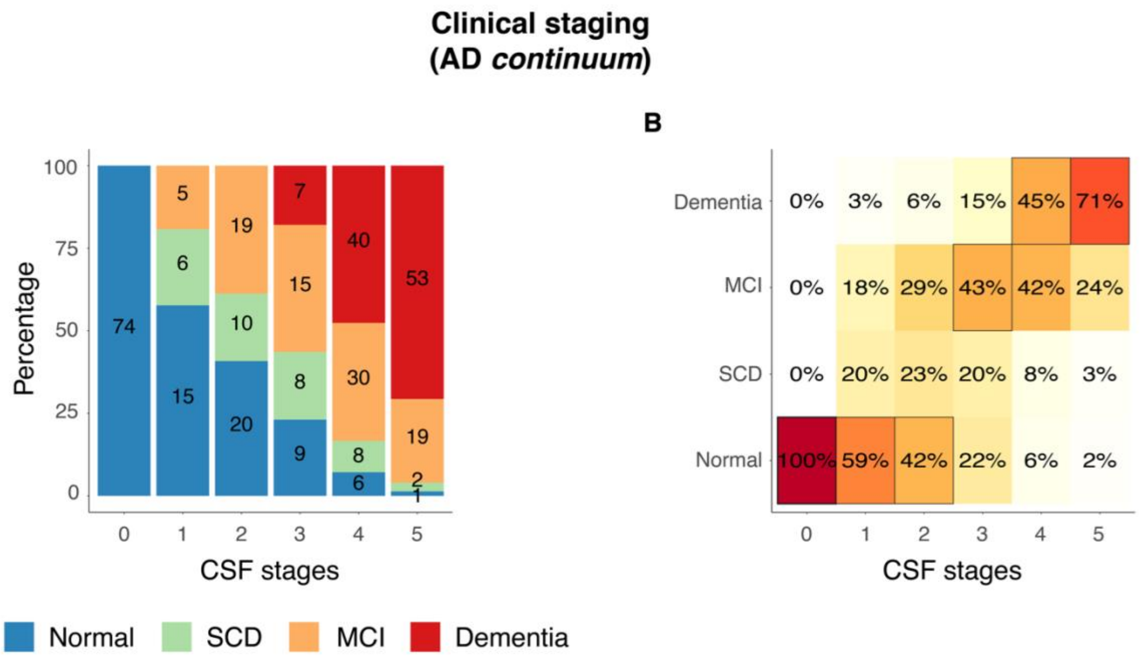

Supplementary Figure 4: CSF stages for predicting clinical stages

Barplots represent the number of participants in each category per CSF stage (A). Numbers of participants in each category per CSF stage are shown within the barplots. An ordinal logistic regression was used for predicting clinical stages by CSF stages. The heatmap represent the predicted percentage of participants in each outcome category by CSF stage. The most probable (highest percentage) category by CSF stage is framed in black. Non-AD dementia cases were excluded from the analysis.

Abbreviations: Aβ, amyloid-β; AD, Alzheimer's disease; ADD+, Alzheimer's disease dementia amyloid-positive; CSF, cerebrospinal fluid; CU, cognitively unimpaired; MCI, mild cognitive impairment; SCD, subjective cognitive decline.

	All (n=399)	CU- (n=80)	CU+ (n=76)	MCI+ (n=86)	ADD+ (n=90)	non-AD (n=67)
**Age, years**	71.4 (8.45)	70.7 (9.46)	71.1 (9.30)	71.9 (7.48)	72.6 (6.88)	70.3 (9.24)
**Women, n(%)**	196 (49.1%)	39 (48.8%)	37 (48.7%)	37 (43.0%)	50 (55.6%)	33 (49.3%)
APOE-ε4 **carriership, n(%)[a]**	228 (57.1%)	26 (32.5%)	56 (73.7%)	60 (69.8%)	65 (72.2%)	21 (31.3%)
**Years of education[b]**	12.3 (3.81)	12.0 (3.22)	12.2 (3.40)	12.7 (4.53)	12.0 (3.98)	12.8 (3.66)
**Amyloid-PET rate[c]**	0.27 (0.39)	0.06 (0.19)	0.43 (0.26)	0.36 (0.51)	-	0.20 (0.55)
**Follow-up time amyloid-PET, years**	2.63 (1.01)	2.81 (1.05)	2.72 (0.98)	2.38 (0.96)	-	2.44 (1.13)
**Number follow-ups, amyloid-PET [range]**	2.39 (0.50) [2-4]	2.46 (0.50) [2-3]	2.44 (0.53) [2-4]	2.29 (0.46) [2-3]	-	2.33 (0.58) [2-3]
**Tau-PET rate[d]**	0.55 (0.98)	0.04 (0.22)	0.27 (0.43)	0.93 (1.19)	1.45 (1.30)	0.13 (0.35)
**Follow-up time tau-PET, years**	2.46 (0.97)	2.80 (1.07)	2.88 (1.02)	2.40 (0.91)	1.73 (0.30)	2.07 (0.53)
**Number follow-ups, tau-PET [range]**	2.50 (0.68) [2-4]	2.59 (0.67) [2-4]	2.70 (0.71) [2-4]	2.57 (0.79) [2-4]	2.23 (0.43) [2-3]	2.18 (0.39) [2-3]
**Cortical thickness rate[e]**	0.258 (0.374)	0.041 (0.167)	0.101 (0.195)	0.293 (0.285)	0.716 (0.360)	0.248 (0.501)
**Follow-up time cortical thickness, years**	2.43 (0.93)	2.77 (1.03)	2.85 (0.98)	2.40 (0.89)	1.72 (0.31)	2.03 (0.44)
**Number follow-ups, cortical thickness [range]**	2.51 (0.66) [2-4]	2.61 (0.64) [2-4]	2.71 (0.72) [2-4]	2.60 (0.76) [2-4]	2.26 (0.45) [2-3]	2.14 (0.36) [2-3]
**mPACC rate[f]**	-0.39 (0.79)	0.05 (0.27)	0.07 (0.41)	0.41 (0.58)	1.22 (1.21)	0.51 (0.87)
**Follow-up time mPACC, years**	2.55 (0.94)	2.78 (1.01)	2.99 (0.87)	3.00 (0.84)	2.03 (0.58)	1.91 (0.70)
**Number follow-ups, mPACC[range]**	2.95 (0.85) [2-5]	2.43 (0.50) [2-3]	3.20 (1.08) [2-5]	3.65 (0.84) [2-5]	2.78 (0.42) [2-5]	2.64 (0.60) [2-5]

Supplementary Table 11: Characteristics of BioFINDER-2 participants with follow-up AD biomarkers

Data is shown as mean (SD) unless otherwise stated. All rates of change are given as z-scores and in all cases higher values represent higher abnormality.

[a], 1 participant missing; [b], 4 participants missing; [c], 181 participants missing; [d], 87 participants missing; [e], 99 participants missing; [f], 57 participants missing.

Abbreviations: Aβ, amyloid-β; AD, Alzheimer's disease; ADD+, Alzheimer's disease dementia amyloid positive; CU-, cognitively unimpaired amyloid negative; CU+, cognitively unimpaired amyloid positive; CSF, cerebrospinal fluid; MCI+, mild cognitive impairment amyloid positive; mPACC, modified preclinical Alzheimer's cognitive composite; nonAD, non-Alzheimer's related disease; PET, positron emission tomography; SD, standard deviation; SUVR, standardized uptake value ratio.

Biomarker	Mean (SD) CSF stage 0	Mean (SD) CSF stage 1	Mean (SD) CSF stage 2	Mean (SD) CSF stage 3	Mean (SD) CSF stage 4	Mean (SD) CSF stage 5
Amyloid-PET	0.03 (0.17)[b,c,d,e]	0.36 (0.30)[a,c]	0.56 (0.25)[a,b,e,f]	0.44 (0.27)[a]	0.36 (0.33)[a,c]	0.23 (0.54)[c]
Tau-PET	0.05 (0.24)[d,e,f]	0.15 (0.27)[d,e,f]	0.18 (0.46)[d,e,f]	0.46 (0.75)[a,b,c,e,f]	1.38 (1.25)[a,b,c,d]	1.18 (1.25)[a,b,c,d]
Cortical thickness	0.09 (0.29)[d,e,f]	0.13 (0.38)[d,e,f]	0.07 (0.18)[d,e,f]	0.30 (0.35)[a,b,c,e,f]	0.44 (0.39)[a,b,c,d]	0.55 (0.35)[a,b,c,d]
mPACC	0.16 (0.50)[d,e,f]	0.05 (0.41)[d,e,f]	0.13 (0.66)[d,e,f]	0.40 (0.66)[a,b,c,e,f]	0.80 (0.91)[a,b,c,d]	0.83 (1.12)[a,b,c,d]

Supplementary Table 12: Statistics of AD-biomarkers longitudinal rates of change and their differences by CSF stage

Mean(SD) z-scores by CSF stages are shown in calculated from a group of cognitively unimpaired amyloid negative participants. Differences among CSF stages were calculated using pairwise Wilcoxon test. mPACC and cortical thickness z-scores were inverted to obtain higher values for higher abnormality. P-values were FDR-corrected for multiple comparisons.

Abbreviations: Aβ, amyloid-β; CSF, cerebrospinal fluid; FDR, false-discovery rate; mPACC, modified preclinical Alzheimer's cognitive composite; PET, positron emission tomography; SD, standard deviation.

	HR [95%CI]	p
**BioFINDER-2**		
CU & MCI to AD dementia	5.8 [2.4 – 14.3]	<0.001
MCI to AD dementia	4.5 [1.8 – 10.8]	<0.001
CU to MCI	33.1 [6.5 - 169.0]	<0.001
**Knight-ADRC**		
CDR=0 & CDR=0.5 to CDR≥1	10.4 [4.2 – 25.6]	<0.001
CDR=0 to CDR≥0.5	6.4 [3.1 – 13.2]	<0.001

Supplementary Table 13: CSF stages for predicting disease progression

Hazard ratios for predicting disease progression for higher CSF stages (4-5) compared to lower positive CSF stages (1-3). Models were adjusted for age and sex in all cases, and additionally disease status at baseline (*i.e.*, CU/MCI in BioFINDER-2 or CDR=0/0.5 in Knight-ADRC), if appropriate.

Abbreviations: AD, Alzheimer's disease; CDR, clinical dementia rating; CU, cognitively unimpaired; CSF, cerebrospinal fluid; MCI, mild cognitive impairment.

CSF stage at baseline	All (n=51)	CU- (n=37)	CU+ (n=11)	Other dementias (n=3)
**Age, years**	69.0 (6.5)	68.1 (6.4)	71.5 (6.9)	70.1 (6.0)
**Women, n(%)**	25 (49.0%)	17 (45.9%)	6 (54.5%)	2 (66.7%)
***APOE-ε4* carriershp, n(%)**	18 (35.3%)	9 (24.3%)	8 (72.7%)	1 (33.3%)
**Years of education**	16.2 (2.32)	16.0 (2.48)	16.5 (1.51)	17.3 (3.06)
**Years between baseline and follow-up**	2.84 (0.73)	2.91 (0.72)	2.62 (0.86)	2.88 (0.12)
**CSF stage at baseline, n(%)**				
**0**	38 (74.5%)	36 (97.3%)	0 (0%)	2 (66.7%)
**1**	1 (2.0%)	0 (0%)	1 (9.1%)	0 (0%)
**2**	7 (13.7%)	1 (2.7%)	5 (45.5%)	1 (33.3%)
**3**	4 (7.8%)	0 (0%)	4 (36.4%)	0 (0%)
**4**	0 (0%)	0 (0%)	0 (0%)	0 (0%)
**5**	1 (2.0%)	0 (0%)	1 (9.1%)	0 (0%)
**CSF stage at follow-up, n(%)**				
**0**	38 (74.5%)	37 (97.4%)	1 (100%)	0 (0%)
**1**	0 (0%)	0 (0%)	0 (0%)	0 (0%)
**2**	6 (11.8%)	1 (2.6%)	0 (0%)	5 (71.4%)
**3**	4 (7.8%)	0 (0%)	0 (0%)	1 (14.3%)
**4**	2 (3.9%)	0 (0%)	0 (0%)	1 (14.3%)
**5**	1 (2.0%)	0 (0%)	0 (0%)	0 (0%)

Supplementary Table 14: Characteristics of Knight-ADRC participants with follow-up CSF data

Data is shown as mean (SD) unless otherwise stated. For those Knight-ADRC with more than one longitudinal visit we took the one more distant from the baseline.

Abbreviations: CU-, cognitively unimpaired amyloid negative; CSF, cerebrospinal fluid; MCI+, mild cognitive impairment amyloid positive; SD, standard deviation.

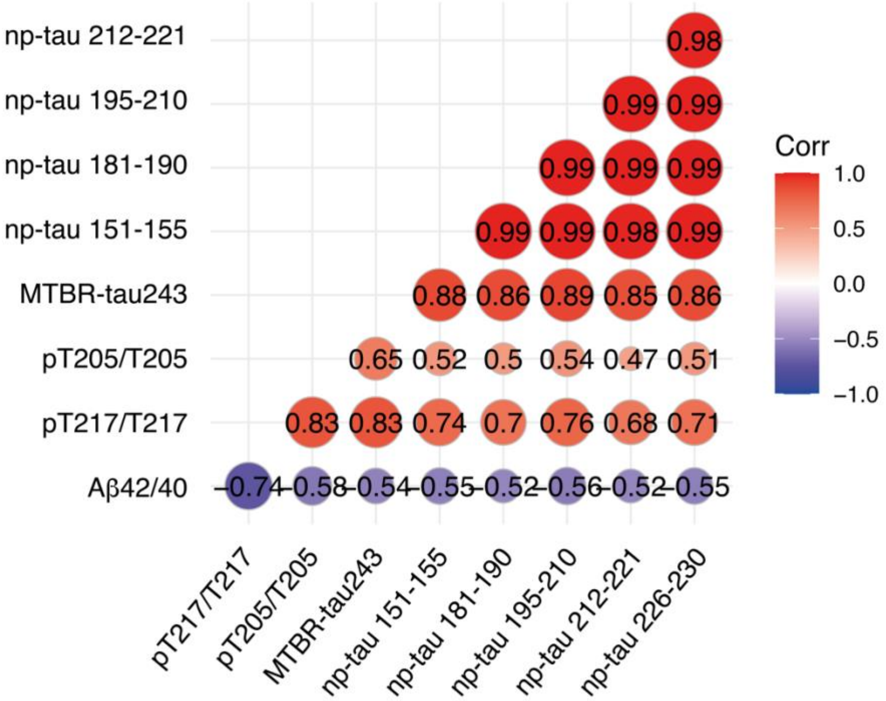

Supplementary Figure 5: Cross-correlation among biomarkers

Cross-correlation among biomarkers included in the final model and all the np-tau fragments at different residues. Pearson's r are shown in the figure.

Abbreviations: Aβ, amyloid-β; MTBR, microtubule binding region; np-tau, non-phosphorylated mid-region tau; pT, phosphorylated tau.

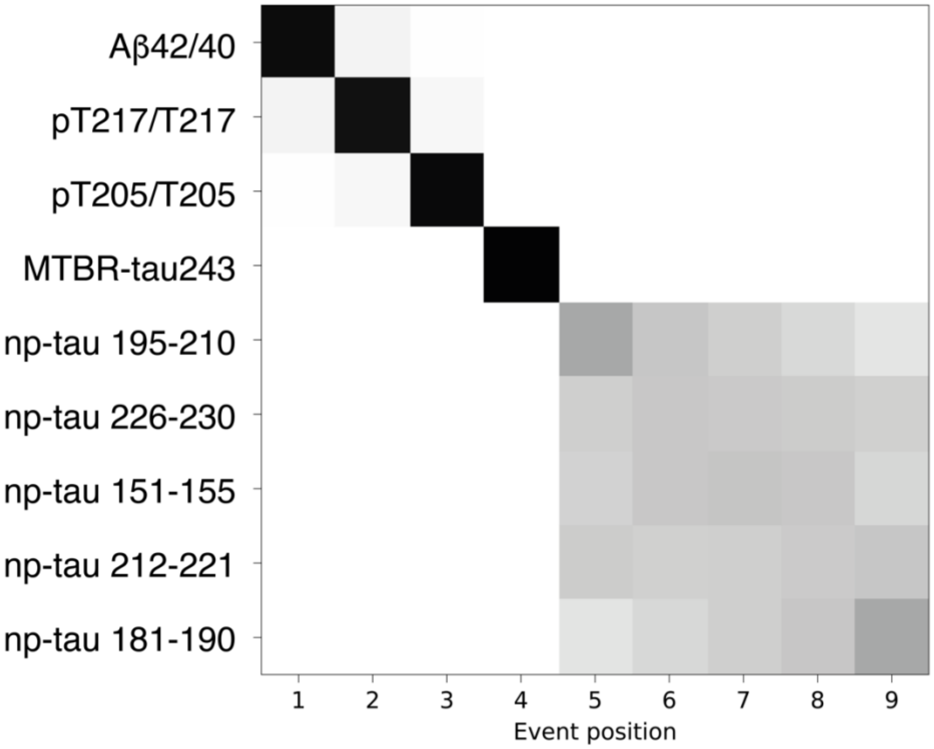

Supplementary Figure 6: Confusion matrix of the ordering of the model when all np-tau fragments are included

Cross-validated confusion matrix of the of the model is shown when including all biomarkers from the final model and all np-tau fragments. Biomarkers are sorted by the time they become abnormal based on the results of SuStaIn. Darkness represents the probability of that biomarker of becoming abnormal at that position, with black being 100%. Thus, the light grey colors seen in all the np-tau fragments represent a low confidence in their ordering.

Abbreviations: Aβ, amyloid-β; MTBR, microtubule binding region; np-tau, non-phosphorylated mid-region tau; pT, phosphorylated tau.

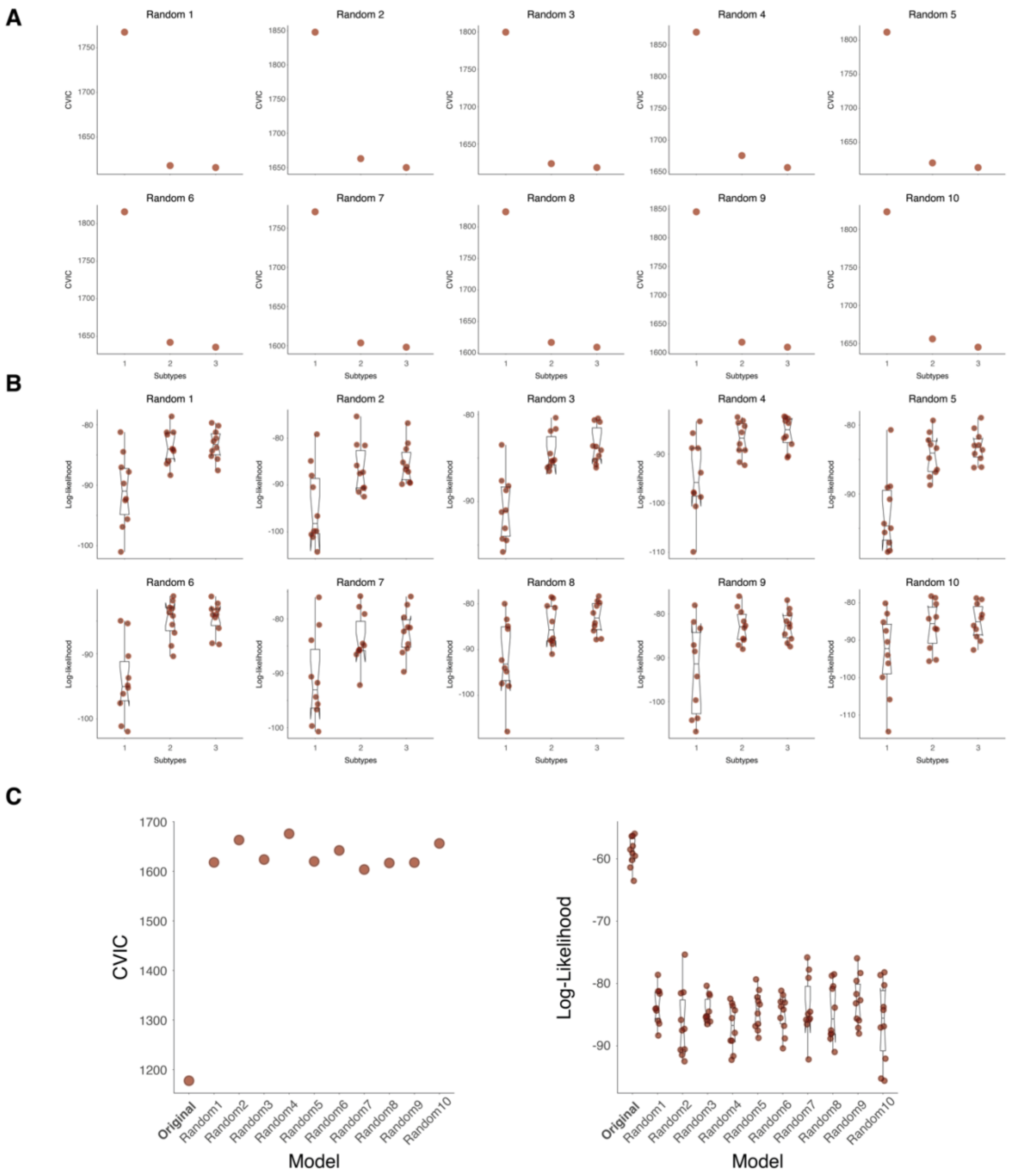

Supplementary Figure 7: Creation of the model in ten random samples

Figure A shows CVIC for the ten random samples created for 1, 2 and 3 subtypes. The lower CVIC in subtypes 2 and 3 suggest that these are performing better than a single subtype model. Figure B shows log-likelihood for the ten cross-validation samples for each subtype in each random sample (same as in A). The higher log-likelihood of 2 and 3 subtypes suggest a better performance of these models than the single subtype model. Based on the criteria of selecting the simplest model for similar performances, the 2 subtypes model seems to be the optimal model in all the random samples. Figure C shows that statistics (left: CVIC, right: log-likelihood) from the original model are significantly better than those from the models created with random samples. CVIC from

the original model is lower (better) than those from the random samples. Whereas log-likelihood is higher (better) than those from the random samples. In boxplots, dots represent each of the ten-fold permutations, central band of the boxplot represents the median of the group, the lower and upper hinges correspond to the first and third quartiles, and the whiskers represent the maximum/minimum value or the 1.5 IQR from the hinge, whatever is lower.

Abbreviations: CVIC, cross-validation information criterion.

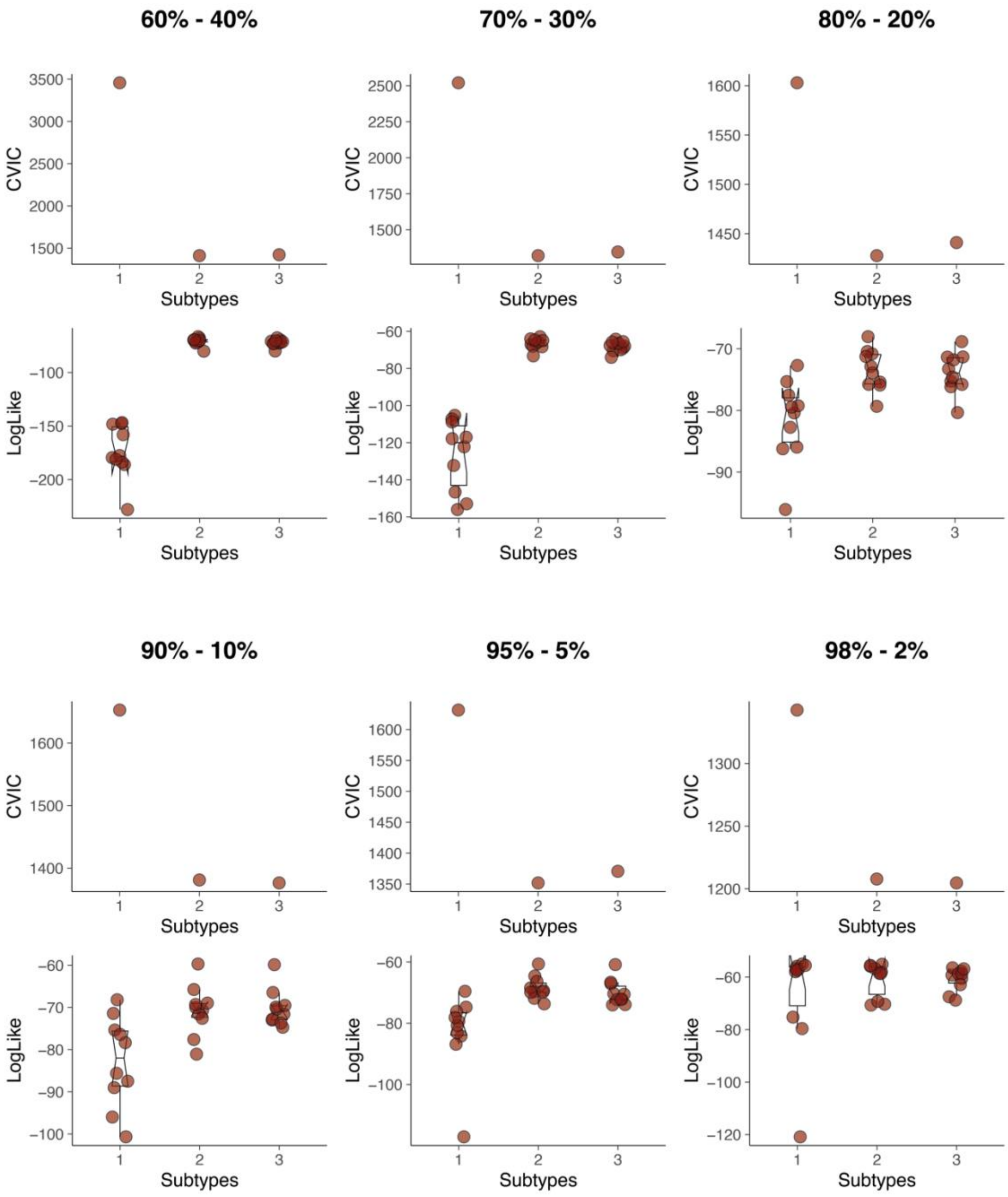

Supplementary Figure 8: Simulation of two subtypes models with decreasing prevalence

SuStaIn results from simulated data with two subtypes ground truth models, when decreasing the prevalence of the smallest subtype. For each prevalence, we show CVIC and log-likelihood, respectively, for the models when forcing one, two or three subtypes in SuStaIn. The lower CVIC in the two-subtypes models suggest that these are the best performing models in all prevalences tested. This is supported by the higher log-likelihood of these same models, up until the prevalence of the smallest subtype is 5%.

In boxplots, dots represent each of the ten-fold permutations, central band of the boxplot represents the median of the group, the lower and upper hinges correspond to the first and third quartiles, and the whiskers represent the maximum/minimum value or the 1.5 IQR from the hinge, whatever is lower.

Abbreviations: CVIC, cross-validation information criterion.

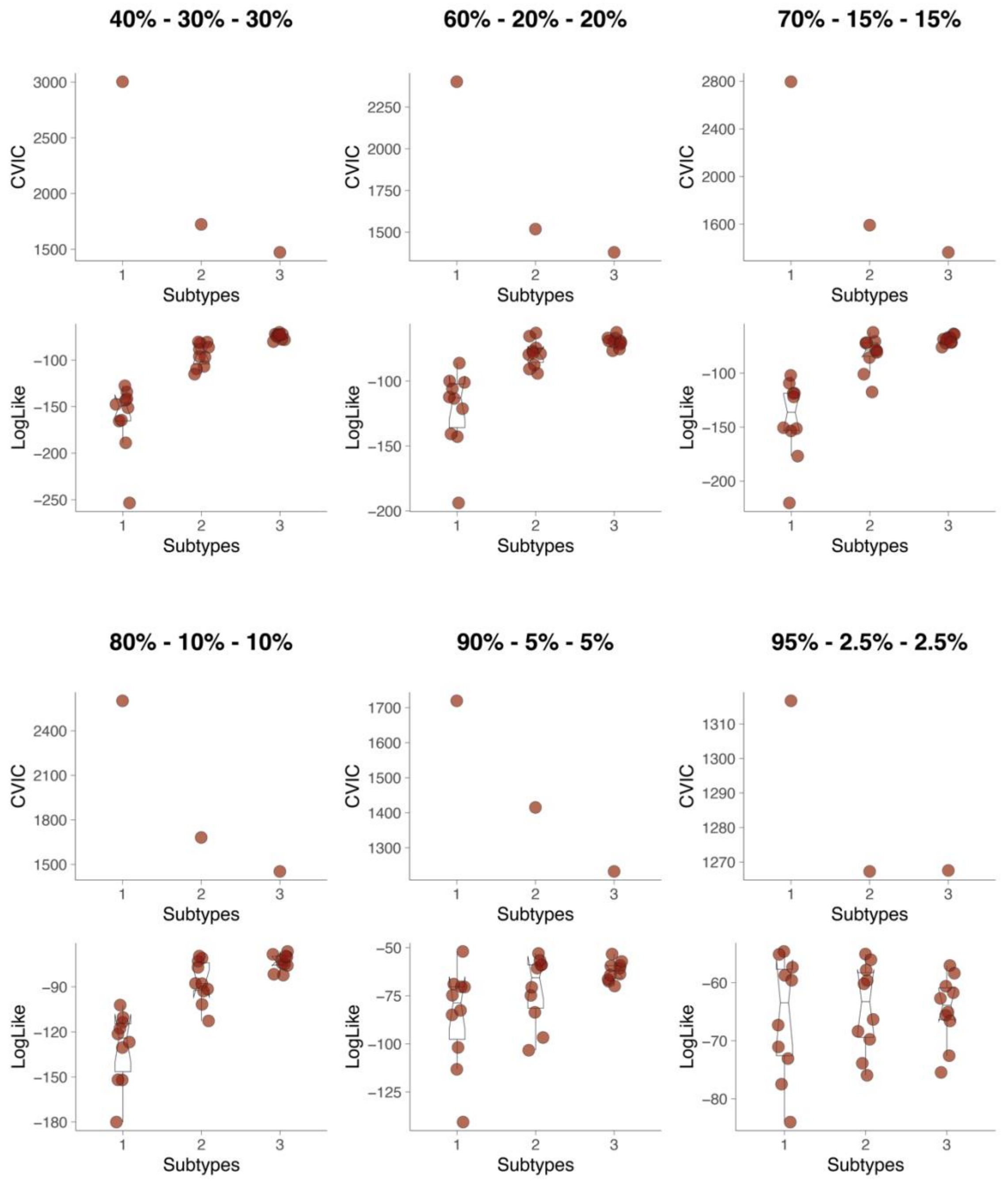

Supplementary Figure 9: Simulation of three subtypes models with decreasing prevalence

SuStaIn results from simulated data with three subtypes ground truth models, when decreasing the prevalence of the two smallest subtypes. For each prevalence, we show CVIC and log-likelihood, respectively, for the models when forcing one, two or three subtypes in SuStaIn. The lower CVIC in the three-subtypes models suggest that these are the best performing models in all prevalence tested until the smaller subtypes are 5%. This is supported by the higher log-likelihood of these same models, up until the

prevalence of the same prevalence. In boxplots, dots represent each of the ten-fold permutations, central band of the boxplot represents the median of the group, the lower and upper hinges correspond to the first and third quartiles, and the whiskers represent the maximum/minimum value or the 1.5 IQR from the hinge, whatever is lower.

Abbreviations: CVIC, cross-validation information criterion.

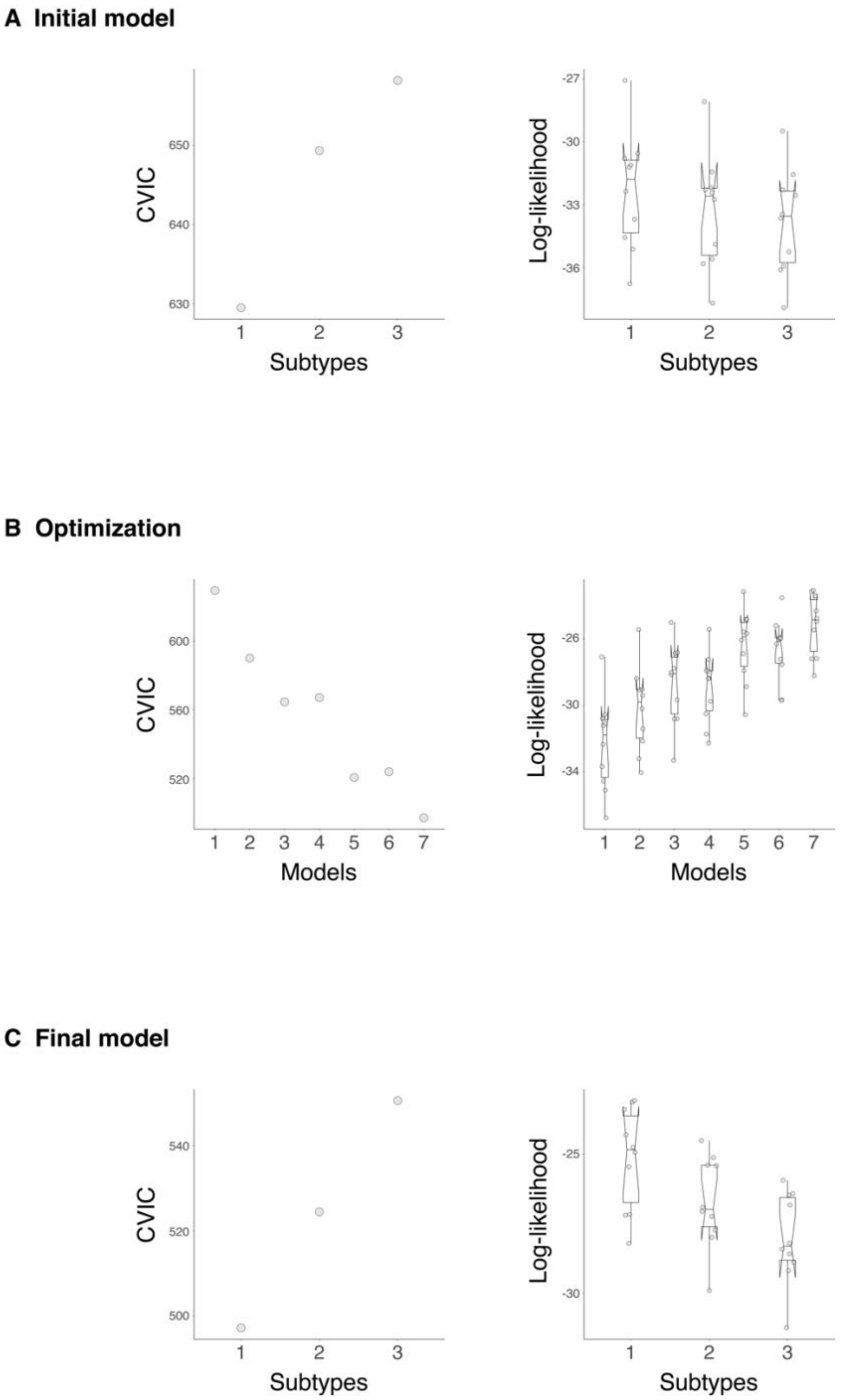

Supplementary Figure 10: Creation and optimization of the model in the Knight ADRC cohort

Initial model with all CSF biomarkers (Aβ42/40, pT217/T217, pT231/T231, pT181/T181, pT205/T205, MTBR-tau243 and np-tau) is shown in A. The two columns represent CVIC and log-likelihood, of this model for one, two and three subtypes. Each dot in log-likelihood plot represents one of the ten cross-validation sets of data. Lower CVIC and higher log-likelihood values represent better performance of the model. Thus, one subtype is complex enough to explain the variability observed in the data (A and C).

Given that some biomarkers (pT217/T217, pT231/T231 and pT181/T181) show high overlap on the ordering, we optimized the model by removing these biomarkers systematically (B). All models without one or two of these biomarkers were tested (models 2 to 7). CVIC (left) and cross-validated confusion matrixes (right) for each of these models are shown in B, respectively. CVIC shows that the optimal model was that excluding both pT231/T231 and pT181/T181 (model 7). Both CVIC and log-likelihood measures show that one subtype was the optimal model when using this set of biomarkers (C). In boxplots, dots represent each of the ten-fold permutations, central band of the boxplot represents the median of the group, the lower and upper hinges correspond to the first and third quartiles, and the whiskers represent the maximum/minimum value or the 1.5 IQR from the hinge, whatever is lower.

Abbreviations: Aβ, amyloid-β; CVIC, cross-validation information criterion; MTBR, microtubule binding region; pT, phosphorylated tau; SuStaIn, subtype and stage inference.

REFERENCES

1. Young, A. L. *et al.* Ordinal SuStaIn: Subtype and Stage Inference for Clinical Scores, Visual Ratings, and Other Ordinal Data. *Front Artif Intell* **4**, (2021).
2. Ossenkoppele, R. *et al.* Amyloid and tau PET-positive cognitively unimpaired individuals are at high risk for future cognitive decline. *Nat Med* **28**, 2381–2387 (2022).
3. Pontecorvo, M. J. *et al.* A multicentre longitudinal study of flortaucipir (18F) in normal ageing, mild cognitive impairment and Alzheimer’s disease dementia. *Brain* **142**, 1723–1735 (2019).
4. Mintun, M. A. *et al.* Donanemab in Early Alzheimer’s Disease. *New England Journal of Medicine* **384**, 1691–1704 (2021).
