# Supplementary material for: Disease staging of Alzheimer’s disease using a CSF-based biomarker model
Source: Nat Aging. 2024 Mar 21;4(5):694–708. doi: 10.1038/s43587-024-00599-y (PMC11108782; doi:10.1038/s43587-024-00599-y)
Supplement: Supplementary file 2 — Reporting Summary [file 43587_2024_599_MOESM2_ESM.pdf]

nature portfolio

Corresponding author(s): Oskar Hansson
Gemma Salvadó

Last updated by author(s): Feb 8, 2024

Reporting Summary

Nature Portfolio wishes to improve the reproducibility of the work that we publish. This form provides structure for consistency and transparency in reporting. For further information on Nature Portfolio policies, see our Editorial Policies and the Editorial Policy Checklist.

Statistics

For all statistical analyses, confirm that the following items are present in the figure legend, table legend, main text, or Methods section.

n/a	Confirmed	
☐	☒	The exact sample size (*n*) for each experimental group/condition, given as a discrete number and unit of measurement
☐	☒	A statement on whether measurements were taken from distinct samples or whether the same sample was measured repeatedly
☐	☒	The statistical test(s) used AND whether they are one- or two-sided<br>*Only common tests should be described solely by name; describe more complex techniques in the Methods section.*
☐	☒	A description of all covariates tested
☐	☒	A description of any assumptions or corrections, such as tests of normality and adjustment for multiple comparisons
☐	☒	A full description of the statistical parameters including central tendency (e.g. means) or other basic estimates (e.g. regression coefficient) AND variation (e.g. standard deviation) or associated estimates of uncertainty (e.g. confidence intervals)
☐	☒	For null hypothesis testing, the test statistic (e.g. *F*, *t*, *r*) with confidence intervals, effect sizes, degrees of freedom and *P* value noted<br>*Give P values as exact values whenever suitable.*
☒	☐	For Bayesian analysis, information on the choice of priors and Markov chain Monte Carlo settings
☒	☐	For hierarchical and complex designs, identification of the appropriate level for tests and full reporting of outcomes
☐	☒	Estimates of effect sizes (e.g. Cohen's *d*, Pearson's *r*), indicating how they were calculated

Our web collection on statistics for biologists contains articles on many of the points above.

Software and code

Policy information about availability of computer code

Data collection	No software was used.
Data analysis	R version 4.1.0 was used for comparison analyses. The main packages used were pROC and cutpointr for ROC analyses, stats for linear regression models, MASS and lmr for logistic regression models, survival and survminer for Kaplan-Meier curves, and ggplot2 for creating plots. PySuStaIn (downloaded 08/2022) was used for creating the CSF staging model. FreeSurfer (v.6.0.) was used to parcelate MRI.

For manuscripts utilizing custom algorithms or software that are central to the research but not yet described in published literature, software must be made available to editors and reviewers. We strongly encourage code deposition in a community repository (e.g. GitHub). See the Nature Portfolio guidelines for submitting code & software for further information.

Data

Policy information about availability of data

All manuscripts must include a data availability statement. This statement should provide the following information, where applicable:

- Accession codes, unique identifiers, or web links for publicly available datasets
- A description of any restrictions on data availability
- For clinical datasets or third party data, please ensure that the statement adheres to our policy

For BioFINDER-2 data, anonymized data will be shared by request from a qualified academic investigator for the sole purpose of replicating procedures and results presented in the article and as long as data transfer is in agreement with EU legislation on the general data protection regulation and decisions by the Ethical

Review Board of Sweden and Region Skåne, which should be regulated in a material transfer agreement. For Knight ADRC data are available to qualified investigators who have a proposal approbed by an institutional committee (https://knightadrc.wustl.edu/Research/ResourceRequest.htm) that meets monthly. the study must be approved by an institutional review board to ensure ethical reseearch practives and investigators must agree to the terms and conditions of the data use agreement, which includes not distributing the data without permission. Contact persons are Oskar Hansson and Randall J Bateman, respectively. After contacting the person, they will respond within a month.

Human research participants

Policy information about studies involving human research participants and Sex and Gender in Research.

Reporting on sex and gender	Sex is reported in the the descriptive tables.
Population characteristics	Please see main tables of the manuscript.
Recruitment	Recruitment is described in the manuscript, and on www.clinicaltrials.gov for NCT03174938 (BioFINDER-2).
Ethics oversight	The Swedish Ethical Review Authority and Washington University Human Research Protection Office

Note that full information on the approval of the study protocol must also be provided in the manuscript.

Field-specific reporting

Please select the one below that is the best fit for your research. If you are not sure, read the appropriate sections before making your selection.

☒ Life sciences ☐ Behavioural & social sciences ☐ Ecological, evolutionary & environmental sciences

For a reference copy of the document with all sections, see nature.com/documents/nr-reporting-summary-flat.pdf

Life sciences study design

All studies must disclose on these points even when the disclosure is negative.

Sample size	The study included two prospective studies (BioFINDER-2 [n=426] and Knight ADRC [n=222]) with large sample sizes. All participants with available CSF measures at baseline were analyzed in this study.
Data exclusions	Only extreme outliers in longitudinal analyses were excluded. Excluded outliers are detailed in the text.
Replication	We replicated key findings in two large independent cohorts (BioFINDER-2 and Knight ADRC) with significant differences in demographics and outcome measures.
Randomization	There was no randomization in this study. We did not perform any adjustment for covariates due to the comparison of biomarkers within the same sample.
Blinding	CSF analyses were performed by individuals who were blinded to the clinical data. Co-authors who performed the data preprocessing were blinded to demographic and clinical characteristics of individuals.

Reporting for specific materials, systems and methods

We require information from authors about some types of materials, experimental systems and methods used in many studies. Here, indicate whether each material, system or method listed is relevant to your study. If you are not sure if a list item applies to your research, read the appropriate section before selecting a response.

Materials & experimental systems

n/a	Involved in the study	
☐	☒	Antibodies
☒	☐	Eukaryotic cell lines
☒	☐	Palaeontology and archaeology
☒	☐	Animals and other organisms
☐	☒	Clinical data
☒	☐	Dual use research of concern

Methods

n/a	Involved in the study	
☒	☐	ChIP-seq
☒	☐	Flow cytometry
☒	☐	MRI-based neuroimaging

Antibodies

Antibodies used	Tau1 (generated by Drs Nicholas Kanaan) and HJ series (HJ8.5, HJ8.7, HJ32.11 and HJ34.8) antibodies (generated by Dr. David Holtzman) were used. Detailed information of the immunoassays in the manuscript has been published previously (and is referred to in the manuscript).
Validation	Tau1, HJ8.5 and HJ8.7 were validated in the following studies:<br>-Barthélemy NR, et al. Site-specific cerebrospinal fluid tau hyperphosphorylation in response to Alzheimer's disease brain patholgy: Not all tau phospho-sites are hyperphosphorylated. Journal of Alzheimer's disease, 2022, 85(1): 415-29.<br>-Sato, et al. Tau kinetic in neurons and the human central nervous sytem. Neuron 2018. 98(4): 861-4.<br>HJ32.11 and HJ34.8 were newly generated antibodies and we confirmed that immunoprecipitation procedures using there antibodies worked well by the two replicate cohorts analyses.

Clinical data

Policy information about clinical studies

All manuscripts should comply with the ICMJE guidelines for publication of clinical research and a completed CONSORT checklist must be included with all submissions.

Clinical trial registration	BioFINDER-2 study was registered at www.clinicaltrials.gov for NCT03174938 .
Study protocol	Please see www.clinicaltrials.gov for the outlines of NCT03174938 (BioFINDER-2) .
Data collection	BioFINDER-2 data are collected at the memory clinics of Skåne University Hospital and Ängelholm's hospital in Sweden. Participants in Knight ADRC cohort were community-dwelling volunteers enrolled in studies of memory and aging.
Outcomes	The primary outcome is CSF stages determined by SuStaIn using CSF biomarkers abnormalities. These stages were then compared to amyloid- and tau-PET, neurodegeneration and cognitive measures, both cross-sectional and longitudinally. CSF stages were also used to predict amyloid- and tau-PET status (positive/negative), diagnosis, and clinical progression.
